# Signatures of the Systemic Effects of a Snake Venom and Antivenom: Multiomics Profiling of the Kidney Pathology

**DOI:** 10.1016/j.mcpro.2025.101023

**Published:** 2025-06-27

**Authors:** Alison F.A. Chaves, Bianca C.S.C. de Barros, Miguel Cosenza-Contreras, Mariana S.L.C. Morone, Ana T.A. Sachetto, Niko Pinter, Marlene Schmid, Marcelo L. Santoro, Oliver Schilling, Solange M.T. Serrano

**Affiliations:** 1 Laboratory of Applied Toxinology, Center of Toxins, Immune-Response and Cell Signaling (CeTICS), Butantan Institute, São Paulo, Brazil; 2 Institute of Clinical Pathology, University Medical Center Freiburg, Freiburg, Germany; 3 Central Animal Facility, Butantan Institute, São Paulo, Brazil

**Keywords:** *Bothrops jararaca*, mouse kidney, N-terminomics, phosphoproteomics, proteomics, snake venom

## Abstract

Animal venoms comprise many toxins that work in concert to break apart the robust homeostatic systems of prey organisms. Conversely, prey organisms actively antagonize each step of envenoming, which displays a complex kinetics involving important changes at molecular, cell, tissue, and organism levels. In this study we explored the mammalian host response to envenoming using proteomics/N-terminomics and phosphoproteomics approaches to evaluate the *in vivo* effects of *Bothrops jararaca* venom in the mouse kidney after injection in the thigh muscle (1.6 mg/kg), mimicking a snakebite, and the impact of anti-*Bothrops* antivenom injected 1 h later (1.6 mg/kg; i.v. tail). For proteomics/N-terminomics, proteins were TMT-labeled, to allow for specific (tryptic) and semi-specific searches of MS/MS spectra to assess both global proteome and degradome. We quantified >7000 proteins, and prominent changes were observed in the kidney tissue, where protein differential abundance was identified after 3, 6, and 24 h, including markers of acute-phase response and injury. Likewise, the N-terminomic analysis revealed a significant impact of venom progressing from 3 h to 24 h, resulting in dysregulated proteolysis and indicating the activation of host proteases. The protease fingerprint matched legumain and cathepsin profiles. Venom toxins also promoted alteration in the dynamics of phosphorylation, with the activation of kinases. Under the conditions tested, antivenom administration (i) did not reduce the number of differentially abundant proteins and inflammation markers, (ii) partially attenuated the generation of proteolytic products in envenomed animals, and (iii) directly perturbed the phosphorylation signaling in control animals. Taken together, our findings underscore for the first time the mouse renal response to a protease-rich venom, revealed by the dynamic alteration in protein abundance, protease targets, and phosphorylation events, providing new facets of snake venom and antivenom systemic effects, which are important for the development of new therapies.

## Highlights

• Kidney histopathology did not show alterations up to 24 h after *Bothrops jararaca* venom injection in the thigh muscle.
• Proteomics detected altered levels of inflammatory markers in the kidneys.
• N-terminomic analysis revealed a major role of endogenous proteases in venom-triggered proteolysis.
• Phosphoproteomic analysis showed altered MAPK activity and inflammatory signaling.
• Anti-*Bothrops* antivenom partially mitigated proteolysis and affected phosphorylation.

## Graphical Abstract

**Figure:**
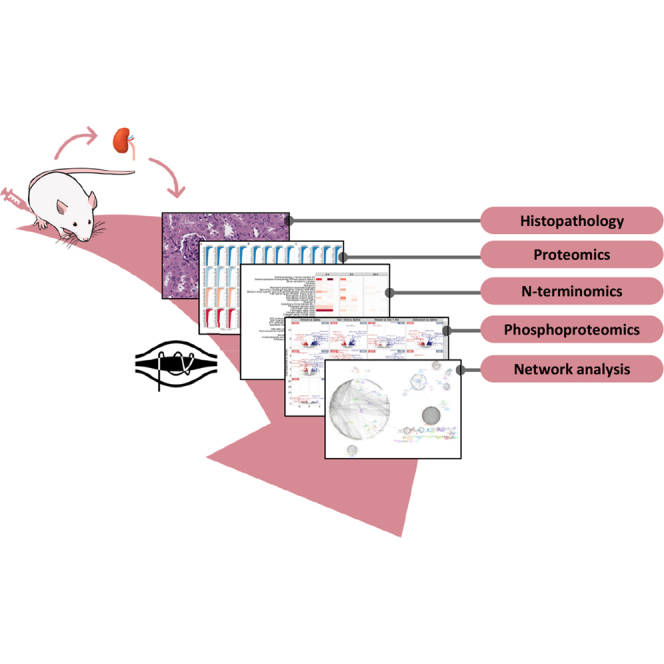

## In Brief

Snake envenomation is a significant health issue worldwide, particularly in rural, low-income regions, and is recognized by the World Health Organization as a neglected tropical disease. Clinical manifestations of human envenomation by snakebite involve local damage and systemic effects. *Bothrops jararaca* is one of the most abundant snake species in Brazil, inhabiting tropical forests and areas of more open vegetation. Due to its wide geographic distribution, it represents an important portion of snakebites, especially in the densely populated regions of Southeastern Brazil. Here we applied multiomics technologies to explore *B. jararaca* venom systemic effects after injection in the mouse thigh muscle, mimicking an event of snakebite, and the impact of antivenom treatment. The findings of this proteomic/degradomic/phosphoproteomic study underscore for the first time the mouse renal response to a protease-rich venom, revealed by the dynamic alteration of protein abundance, protease targets and phosphorylation events, providing new facets of snake venom and antivenom systemic effects, which are important for the development of new therapies.

Snakebite envenoming is a life-threatening disease resulting from the inoculation of venom into humans through fangs located in the frontal region of the maxillary bones in viperid and elapid snakes, usually in accidental events (1, 2). Around 5.4 million snake bites are estimated to occur each year, resulting in 1.8 to 2.7 million cases of human envenoming, with a wide range of case incidences between different countries (World Health Organization, WHO, 2019). As a result, it is estimated that approximately 81,000 to 138,000 people die each year because of snakebite accidents and that three times as many people suffer amputation or other permanent dysfunction afterwards (https://www.who.int/news-room/fact-sheets/detail/snakebite-envenoming).

Snakes belonging to the *Bothrops* genus are among the most studied neotropical viperids, both in terms of ecological characteristics and venom toxin families. *Bothrops jararaca* is one of the most abundant species in Brazil, inhabiting tropical forests and areas of more open vegetation (3). Due to its wide geographic distribution, it represents an important portion of snakebites, especially in the densely populated regions of Southeastern Brazil, where it is responsible for most accidental envenomation cases (4, 5).

Envenoming by *Bothrops* snakes is characterized by prominent changes at the bite site, including pain, swelling, blisters, hemorrhage, and necrosis, which can result in permanent sequelae such as dysfunction or loss of the affected tissue (6, 7). At the systemic level, the pathological effects of venom include hypotension, hemostatic disorders, gingival bleeding, and in severe cases, circulatory shock and acute renal injury (8, 9, 10, 11). These effects are triggered by venom components belonging to a limited number of protein families, such as snake venom metalloproteases (SVMP), snake venom serine proteases (SVSP), phospholipases A2 (PLA2), type C lectins (CTL), L-aminoacid oxidases (LAAO), and biologically active peptides (12, 13, 14).

Parenteral administration of antivenoms from hyperimmunized animals is the first-choice therapy (15, 16, 17). Local and systemic effects of envenoming develop rapidly after inoculation, making neutralization by antivenoms difficult if carried out late due to limited access to medical care or insufficient antivenoms (1, 5, 18, 19). In Brazil, the antivenom used for the therapy of *Bothrops* accidents is produced at Butantan Institute by hyperimmunization of horses with a mixture of venoms (*B. jararaca*, 50%; *Bothrops jararacussu*, 12.5%; *Bothrops alternatus*, 12.5%; *Bothrops moojeni*, 12.5%; *Bothrops neuwiedi*, 12.5%). The severity of the local effects relies on the inoculated amount of venom, venom composition, and the site of the bite and may result in permanent sequelae (20, 21, 22, 23). Likewise, administration of antivenom has been reported to neutralize venom systemic effects but not efficiently revert local manifestations induced by inflammatory mediators (1, 18, 24, 25, 26, 27, 28).

Proteases are responsible for hydrolyzing proteins to peptides, regulating their function and fate. Likewise, by processing other proteins, they contribute to creating new biological molecules involved in the generation and amplification of molecular signals, cell cycle regulation, and even apoptosis. SVMPs and SVSPs are among the most abundant toxins in viperid venoms and are protagonists in snakebite envenoming. These proteases are variants of normal proteins, as evidenced by P-III SVMPs, which resemble A Disintegrin And Metalloproteases (ADAMs), and SVSPs, which resemble enzymes of the mammalian coagulation cascade (29, 30). In mammals, many snake venom proteolytic enzymes escape inhibition by plasma protease inhibitors, such as α-2-macroglobulin and serpins, and can exert their catalytic activities on various macromolecular substrates. Thus, the pathological consequences of the viperid snakebite directly reflect the complexity of the venom subproteomes of proteases (31, 32).

Most of the functional activities associated with SVMPs are related to dysregulation of hemostasis, where they essentially exert pro- or anticoagulant effects, and tissue damage (29, 33). Other activities commonly associated with SVMPs include apoptotic and pro-inflammatory effects (33, 34, 35). Several SVMPs were characterized as hemorrhagic, that is, capable of producing experimental hemorrhage in mouse and rabbit tissue *via* degradation of extracellular matrix and disruption of capillary integrity (34, 36, 37).

SVSPs are among the best-characterized venom components that target the coagulation cascade, the fibrinolytic and kallikrein-kinin systems, and platelets, causing an imbalance of the mammalian hemostatic system (38, 39). These proteases act upon macromolecular substrates either by causing limited proteolysis or degradation, thereby playing an important role in the severe coagulopathy promoted by snake venoms.

Snake venom PLA2s induce a wide range of pharmacological effects. They hydrolyze the acyl bond at the sn-2 acyl position of phospholipids, generating lysophospholipids and free fatty acids, including arachidonic acid, that act as signaling molecules in many biological processes (40). In snake envenoming, PLA2s are mainly implicated in triggering pain, edema, inflammation, myonecrosis, and hemostatic disturbances (41, 42, 43).

Acute renal injury (AKI) is prevalent in viperid snake envenomation and involves structural damage and loss of renal function. It is related to severe cases of *Bothrops* snakebite and has an impact on the mortality rate. Snake venom-induced AKI is characterized by ischemia as a consequence of hypovolemia and hypoperfusion, release of inflammation mediators, renal vasoconstriction, hemolysis, deposition of fibrin in glomerular capillaries, and rhabdomyolysis, leading to tubular cytotoxicity and necrosis (8, 44, 45, 46). Although previous studies have provided useful insights into kidney pathology, the molecular mechanisms of venom-induced AKI are poorly understood.

Knowing that different components of *Bothrops* venoms can cause distinct pathological effects (hemostasis dysregulation, inflammation, hemorrhage, tissue necrosis, cardiovascular shock, and kidney injury), it is essential to investigate the profile of host response to the alterations evoked by the inoculation of snake venom, which can provide a complete picture of the envenomation process in contrast to the effects of the injection of a purified venom toxin. Here, we applied multiomics technologies to explore *B. jararaca* venom systemic effects after injection in the mouse thigh muscle, mimicking an event of a snakebite. This study intends to contribute to a more general understanding of the mammalian host response to snake venom in the presence or absence of antivenom in a murine model, with a focus on the kidney pathology.

## Experimental Procedures

### Venom and Antivenom

Lyophilized *B. jararaca* venom from Butantan Institute was reconstituted in 150 mM NaCl and 1 mM CaCl_2_ to a 1 mg mL^−1^ stock solution. The concentration was adjusted to inject 50 μl volume. Anti-*Bothrops* antivenom was provided by Butantan Institute at a concentration of 5 mg mL^−1^ (batch 210020) and is composed of heterologous F(ab')_2_ fraction of horse immunoglobulins.

### *In vivo* Experiments

**Fig. 1:**
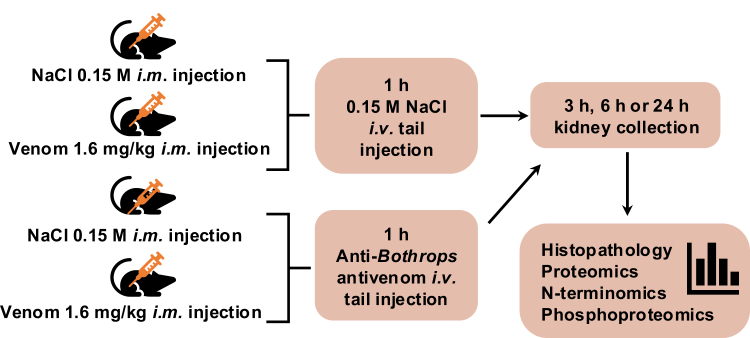
**Schematic diagram of the *in vivo* experimental design.** Groups of four male Swiss mice received an injection of 50 μl 0.15 M NaCl or venom solution (1.6 mg kg^−1^) in the gastrocnemius muscle. One hour later, animals received an injection of 50 μl 0.15 M NaCl or antivenom solution (1.6 mg kg^−1^) in the tail vein. After 3 h, 6 h, and 24 h, the kidneys were collected and preserved for different applications.

Animal procedures were in compliance with good laboratory practices and were approved by the Butantan Institute Ethics Committee on Animal Use (Protocol no. 9991131219). Forty-eight male Swiss mice weighing 24 to 31 g were grouped in 12 groups of four animals each. They were anesthetized with isoflurane (4% for induction and 2.5% for maintenance of general anesthesia) before receiving an inoculum of 50 μl of venom solution (1.6 mg kg^−1^) or 0.15 M NaCl in the right gastrocnemius muscle. After 1 h, mice received an injection of 50 μl 0.15 M NaCl or anti-bothropic antivenom solution (1.6 mg kg^−1^) in the tail vein. Kidneys were collected after 3 h, 6 h, and 24 h, and reserved for formalin fixation (right side) and cryopreservation (left side). For each time point (3 h, 6 h, or 24 h) and each treatment condition, four mice were probed, yielding a total of 12 sets containing four mice each (Fig. 1).

The identification of individual samples was as follows: s = saline (0.15 M NaCl); a = antivenom; v = venom; 3 kn, 6 kn or 24 kn = time in hours after the first injection, to collect the kidneys; a1 – a4 = animal #1, #2, #3 or #4.

For instance:

sa3kna1—mouse 1 (a1) was injected with NaCl (s) *i.m*., 1 h later it received antivenom administration (a) *i.v.*, and had the kidneys collected 3 h after the first injection.

vs6kna2—mouse 2 was injected with venom *i.m.*, 1 h later it received a saline injection *i.v.*, and had the kidneys collected 6 h after the first injection.

va24kna3—mouse 3 was injected with venom *i.m.*, 1 h later it received antivenom injection *i.v.*, and had the kidneys collected 24 h after the first injection.

### Protein Extraction From Cryopreserved Samples for Proteomics/N-Terminomics

A total of 48 samples from mouse kidneys were processed (four samples of each treatment for three different time points). A portion of approximately 10 mg of each tissue was covered with buffer (100 mM HEPES, pH 8.0, 2% SDS, 50 mM EDTA, 100 μg mL^−1^ PMSF, 1 μM E-64, 1 mM Na_4_O_7_P_2_, and 1 mM β-glycerophosphate) for lysis using Precellys Lysing kit CK28-R. The Precellys instrument was set to 3 cycles of 6000 rpm, 30 s, 40 s break, twice with intervals, on ice. Homogenates were centrifuged at 10,000*g* and 4 °C, for 10 min, and the supernatants were carefully transferred to new tubes. Protein content was estimated using BCA assay (Thermo Scientific), and a volume containing 25 μg protein was used in the following reductive alkylation step with 5 mM Tris(2-carboxyethyl)phosphine (TCEP) and 20 mM 2-chloroacetamide (CAA) during 30 min, at room temperature, in the dark.

### TMT-Tagging on Protein Level for Proteomics/N-Terminomics

For isobaric labeling, each of the four animal samples of the four experimental conditions (saline, antivenom, venom and venom + antivenom) totaling 16 samples were subject to the labeling reaction with TMT, in three different groups, according to the three experimental time points (3 h, 6 h, and 24 h). Labeling of protein N-terminal residue was performed using TMTpro 16plex reagent set (Thermo Fisher) by adding isobaric tags in a 1:8 (w/w) protein to TMT reagent ratio (47). A two-step incubation was performed, first for 3 h and 500 rpm at room temperature and then for 20 h at 37 °C and 500 rpm. The reaction was quenched by adding hydroxylamine at 2% final concentration and incubating at 80 °C for 2 h. For LC-MS/MS analysis, three TMT mixes were composed by pooling samples as follows: Mix 1 (16 samples of the 3 h time point), Mix 2 (16 samples of the 6 h time point), and Mix 3 (16 samples of the 24 h time point); and dried by vacuum centrifugation. TMT mixes were resuspended in 200 μl water and acidified with formic acid to pH 2.0.

### Protein Digestion With Trypsin and High-pH Reversed-Phase Chromatography for Proteomics/N-Terminomics

Acidified TMT-labeled protein pools were diluted with six volumes of S-Trap binding buffer (90% methanol, 100 mM Tris-HCl, pH 7.1), loaded on S-Trap columns (Protifi), and centrifuged at 4000*g* for 1 min for protein binding, followed by three washes with 150 μl S-Trap buffer. Digestion buffer (50 mM triethylammonium bicarbonate, pH 8.0) containing trypsin (Serva) at a 1:10 (w/w) enzyme to substrate ratio was loaded into the column and incubated at 47 °C for 2 h, followed by a further incubation step at 37 °C overnight. For peptide elution, three steps of loading and centrifugation were performed (digestion buffer, followed by 0.2% formic acid, then 50%acetonitrile acidified with 0.2% formic acid). Peptides were dried by vacuum centrifugation and stored at −80 °C.

To decrease the peptide complexity and increase proteome coverage, samples were pre-fractionated in an Agilent 1100 HPLC system with an XBRIDGE Peptide BEH C18 column (3.5 μm, 139 Å, 1 × 150 mm), at a flow rate of 40 μl.min^−1^. The elution solvents consisted of 10 mM ammonium formate, pH 10 (buffer A), and 10 mM ammonium formate in 70% acetonitrile, pH 10 (buffer B). Peptides eluted from the S-Trap column were resuspended in 200 μl of buffer A and injected into the column at 30 μl.min^−1^ for 4 min. The column was equilibrated for 60 min at an isocratic flow of 20% buffer B. The fractionation gradient was as follows: 20% to 60% buffer B in 60 min (collecting step), to 100% buffer B within 2 min, and then 1% buffer B for 1 min. A total of 48 fractions were collected and concatenated into 24 peptide fractions (48). Concatenated fractions were dried and resuspended in 0.1% formic acid for LC-MS/MS.

### LC-MS/MS Analysis for Proteomics/N-Terminomics

Peptide fractions were mixed with 400 fmol of indexed Retention Time peptides - iRT (Biognosys) and injected into an EASY-nLC 1200 system equipped with μPAC C18 trapping column (5 μm pillar diameter, 2.5 μm interpillar distance, 2 cm bed length, 100–200 Å pore size) and a μPAC C18 nano-LC analytical column (5 μm pillar diameter, 2.5 μm interpillar distance, 18 μm pillar length, 200 cm bed length, 100–200 Å pore size). For elution, the following gradient was used: 8% to 10% buffer B (80% acetonitrile, 0.1% formic acid) in buffer A (0.1% formic acid) at 700 nl.min^−1^ for 2 min, 10% to 20% buffer B at 350 nl.min^−1^ for 44 min, 20% to 40% buffer B at 350 nl.min^−1^ for 88 min, 55% to 100% buffer B at 350 nl.min^−1^ for 2 min, then 100% buffer B for 40 min. The LC system was coupled to an Orbitrap Elite mass spectrometer (Thermo Fisher) *via* a NanoSpray Flex Ion Source. The instrument was operated in data-dependent acquisition mode for the 10 most intense ions. Scans were acquired at a resolution of 60,000 FWHM at 400 m/z with AGC target set to 10^6^ with profile data type. Precursor selection was obtained using an isolation window of 2 m/z. Most abundant precursor ions were chosen using a scan range from 130 to 1600 m/z and fragmented with higher energy collisional dissociation (HCD). MS2 spectra were analyzed with a resolution of 30,000 FWHM, acquiring in centroid mode. Both MS1 and MS2 were acquired in the Orbitrap mass analyzer. The default charge state was set to 2, rejecting unassigned or single-charge ions. Stepped collisional energy was active, with normalized collision energy set to 37.5 with 2 steps.

### Data Analysis and Statistics of Proteomics/N-Terminomics

Raw files were converted to mzML format using MSConvert (-peak picking filter) and loaded into the FragPipe v21.1. MSFragger v3.7 search engine was configured with a mouse canonical reference FASTA file (mouse-EBI-reference_one-protein-per-gene_2022-04-29 containing 21,995 entries), with decoys and iRT peptide sequences. Peptide N-terminal acetylation (+42.0106) and N-terminal TMT labeling (+304.2071) were specified as variable modifications. TMT labeling at Lys (+304.2071) and carbamidomethylation of Cys (+57.0214) residues were specified as fixed modifications. For evaluation of labeling efficiency, TMTpro16 (+304.2071) on Lys side chains was set as variable modification. ProteinProphet was used to protein inference with a minimum probability of 0.9 and false discovery rate was set to 1%. Precursor and fragment mass tolerance were set to 20 ppm. The ions selected were y, b, and a, considering b∗ as N −17.0265; b0 as N −18.0105; a∗ as N −17.0265; and a0 as N −18.0105. A semi-specific search was conducted using Arg-C as enzyme accepting two missed cleavages. For peptide-spectrum match (PSM) validation, Percolator was used with minimum probability of 0.5 and for deep learning prediction of spectrum features MSBooster was activated. Statistical analysis was performed using R language in the VS Code environment with Quarto markdown documents developed specifically for the data. For the TMT-tagged experiment, differential abundance was inferred using linear model for microarray (limma R package) with robust regression to account for outliers after batch-effect correction (sva R package) and abundance normalization. The ComBat algorithm was applied using the different TMT-mixes as covariate for batch effect correction and protein abundances were normalized to have the same median absolute deviation (MAD). Proteins were considered differentially abundant when the adjusted *p*-value was < 0.05 and the absolute log2 of fold change > |0.58|. Semi-specific peptide cleavage events were analyzed using the TermineR R package for peptide annotation and differentially abundant peptide statistics (47). The essential R packages used for this study were: tidyverse, limma, TermineR, mixOmics, sva, janitor, pheatmap, and corrplot. The network analysis was conducted using Cytoscape version 3.10.2 with stringdb plugin and clusterization with MCODE plugin, while overrepresentation analysis was performed with clueGO plugin for Cytoscape.

### Protein Digestion With Trypsin and Phosphopeptide Enrichment for Phosphoproteomics

The protein digestion was carried out in a Bravo automated liquid handling platform (Agilent Technologies) set to run the SP3 protocol (49). Protein (250 μg) was reduced with 5 mM TCEP and alkylated with 20 mM CAA for 30 min at room temperature, protected from light. A mixture of hydrophobic:hydrophilic magnetic beads (Sera-Mag SpeedBeads, Cytiva) at a 1:1 ratio was washed with water 3 times and resuspended in the original volume. After reduction and alkylation, the samples were incubated at a 1:10 ratio of proteins:beads, added with acetonitrile to a final concentration of 50%, and submitted to 5 min of shaking at 1000 rpm at room temperature. The samples were incubated on a magnetic rack for 5 min, and the liquid volume was completely removed. The beads were washed with 180 μl of 80% acetonitrile 3 times under shaking at 1500 rpm for 5 min between each wash. After the last wash, the beads were resuspended in 50 μl of 100 mM ammonium bicarbonate and incubated overnight with trypsin (Serva) at a 1:50 enzyme to substrate ratio, at 1100 rpm and 37 °C, and then centrifuged for 10 min at 20,000 rpm, at room temperature. The supernatant was carefully transferred to new LoBind tubes, and the peptides were dried by vacuum centrifugation and stored at −80 °C.

The phosphopeptide enrichment was also performed in a Bravo automated liquid handling platform (Agilent Technologies) using the AssayMAP Fe(III) – NTA cartridges. Shortly, 200 μg of tryptic peptides from each sample were loaded onto the IMAC cartridge and processed following the manufacturer's instructions (50). The eluted phosphopeptide samples were vacuum dried and stored at −80 °C.

### LC-MS/MS Analysis for Phosphoproteomics

Phosphopeptide-enriched samples were resolubilized in 0.1% formic acid and analyzed in an Evosep One chromatographic system (Evosep) coupled to a timsTOF fleX (Bruker) mass spectrometer operating in positive mode and acquiring DIA data in a scan range of 400 to 1201 m/z and 0.75 to 1.36 1/k_0_. The isolation width was 26 m/z, and the number of isolation windows was 32, resulting in a total cycle time of 1.81 s, generating ∼7 datapoints per peak. The chromatographic length was set to 30 SPD. Mass tolerance for precursor and fragment ions was 12 ppm at MS1 and MS2.

### Data Analysis and Statistics of Phosphoproteomics

The tdf files (Bruker) were converted to HTRMS format before searching in Spectronaut (version 18.0.230605.50606; Biognosys). The canonical mouse FASTA file (mouse-EBI-reference_one-protein-per-gene_2022-04-29) was used for prediction of the spectral library. The specificity for trypsin was set to two missed cleavages. The peptide length was set to 7 to 52 amino acids. Carbamydomethilation of Cys was selected as fixed modification and N-terminal acetylation, oxidation of Met, and phosphorylation of Ser, Thr, Tyr were selected as variable modifications. The output PTM site report from Spectronaut was filtered to keep phosphopeptides with protein group q-value <0.01 and PTM site probability >0.95. The remaining phosphopeptides were normalized to the protein abundance before quantitative analysis. The regulatory site information was downloaded from PhosphoSitePlus database and integrated into the dataset for further analysis. Sparse partial least squares discriminant analysis implemented in mixOmics R library was used. The phosphopeptide differential abundance was calculated using limma R library.

### Experimental Design and Statistical Rationale

A total of 48 samples from mouse kidney tissue were processed, including four samples of each treatment (saline, antivenom, venom, and venom + antivenom) for three different time points. Labeling of protein N-terminal residue was performed using TMTpro 16plex, and TMT plex designs as described for multiplexed quantification experiments in the TMT-Tagging on Protein Level for Proteomics/N-Terminomics topic of the Experimental Procedures section. High pH-HPLC pre-fractionation of TMT-labeled peptide pools (three mixes composed of 16 samples of each time point), digested with trypsin, resulted in a total of 48 fractions that were concatenated into 24 peptide fractions for each TMT mix, composing a total of 72 LC-MS/MS runs. The Database searching parameters and filtering thresholds for tandem mass spectra are detailed in the Data Analysis and Statistics of Proteomics/N-Terminomics and Phosphoproteomics, respectively. False discovery rate was estimated using the target-decoy approach. All quantified TMT-labeled N-terminal peptides and phosphorylated peptides, including the peptide sequence, precursor charge, and mass/charge, all observed modifications, scores, and relevant quantitation data, are available in the MassIVE repository. For the analysis of global quantitative proteomics and N-terminomics, the psm.tsv files for each TMT mix contain all the quantitative information used to extract the specific and semi-specific peptides, while for the phosphoproteomics analysis the Phospho_Kifney_report_PTMSiteReport (Normal).tsv file contains all the information to extract the phosphopeptides, with localization score and peptide features.

### Histological and Immunohistochemical Analysis of Kidney Tissue

Kidneys were collected after 3 h, 6 h, and 24 h of the first injection and fixed with 3.7% formalin in PBS for 6 h, and later, slices (5 μm thick) of coronal-sectioned tissue were embedded in paraffin by Histocell (São Paulo, Brazil). For deparaffinization and hydration, slides were immersed in xylene for 5 min and washed with a decreasing ethanol gradient (96%, 70%, and 50%) for 1 min each, followed by a wash with ultrapure water for 5 min, and stained with hematoxylin and eosin (H&E) and analyzed by light microscopy.

Slides were also subjected to heat-induced epitope retrieval (HIER) for immunohistochemistry, according to Paulsen *et al.* (51) with some modifications. Briefly, slides were immersed in TEG buffer (10 mM Tris, pH 9.0, containing 0.5 mM EGTA) or citrate buffer (10 mM sodium citrate, pH 6.0) and heated in a microwave oven with 500 W for 12 min. Further, slides were kept immersed in the buffer and cooled at room temperature for 30 min. Finally, samples were washed for 5 min with ultrapure water and subjected to blocking of endogenous peroxidase activity with 10% hydrogen peroxide and 10% methanol in phosphate-buffered saline (PBS), followed by blocking of aldehyde groups with 50 mM ammonium chloride for 30 min at room temperature. Blocking of possible nonspecific binding was performed with PBS containing 1% bovine serum albumin (BSA), 0.2% gelatin and 0.05% saponin for 1 h. Each slide was incubated with a rabbit anti-legumain primary antibody (Cell Signaling #93627) diluted 1:250 for 16 h at 4 °C, followed by incubation with an anti-rabbit IgG horseradish peroxidase (HRP)-conjugated secondary antibody (#926-80011, Li-Cor). Between steps, slides were washed three times with PBS containing 0.1% BSA, 0.2% gelatin, and 0.05% saponin for 10 min. Proteins reactive with the primary antibody were identified by detecting the peroxidase activity by incubating the samples with diaminobenzidine (DAB + substrate kit, ab64238, Abcam) following the manufacturer's instructions. After three washes with PBS and three washes with ultrapure water, slides were counterstained with hematoxylin for 2 min and excess dye was removed with ultrapure water. Finally, 20 μl of Erv-Mount (EasyPath, EP-51-05041) was added to the slides, which were covered with glass coverslips, and analyzed by light microscopy using a Nikon Eclipse Ei microscope (Nikon). The images were acquired with Prime Cam application and processed using the QuPath v.0.4.3 software.

## Results

### Histopathological Analysis Does Not Show Alterations in the Mouse Kidney Morphology After Venom Injection in the Thigh Muscle

**Fig. 2:**
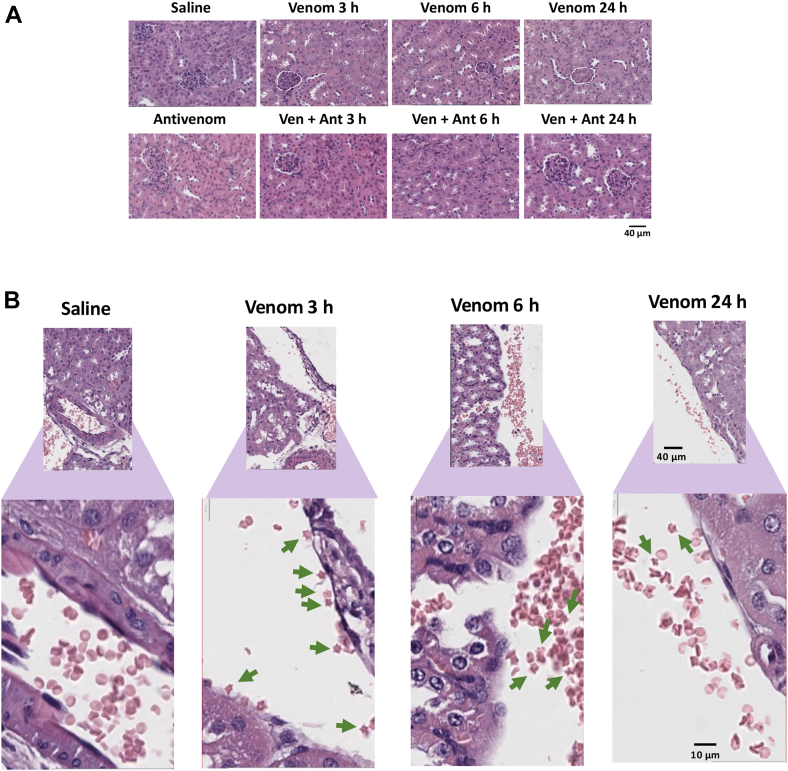
**Histopathological analysis of kidney samples from mice injected with *B*. *jararaca* venom in the thigh (gastrocnemius) muscle.***A*, H&E staining for kidney tissue in mice injected with saline or venom at different time points. *B*, H&E staining of kidney tissue spotting normal erythrocytes in mice which received saline injection and acanthocytes in mice which received venom injection, at different time points. *Green arrows* indicate the presence of acanthocytosis.

After the bloodstream, the kidneys are the main target for the systemic effects of *B. jararaca* snakebite envenomation. In this study, the histopathological evaluation of H&E-stained kidney samples did not show marked morphological changes in the structure of glomeruli and tubules regardless of the time after venom injection or antivenom administration, up to 24 h (Fig. 2*A*). Nevertheless, mice that received venom injection showed acanthocytosis to such a degree that it was possible to detect the presence of acanthocytes in kidney samples (Fig. 2*B*). This morphological alteration in red blood cells was not observed in control samples, ruling out the possibility that it could be an artifact of sample preparation. Our results showed the presence of acanthocytes from 3 h of venom injection; however, acanthocytosis spontaneously resolved after 24 h, regardless of antivenom administration.

### Peptide sample pre-fractionation combined with a long gradient enables high-quality proteomics/N-terminomics results on an Orbitrap Elite platform with OT/OT acquisition

For quantitative, TMT-based proteomic/N-terminomic analysis of kidney tissue, different strategies were tested for optimizing peptide fraction concatenation and gradient length, using samples of animals injected with saline. Two concatenation strategies were tested to cover a wide separation window, pooling either two or four fractions of a total of 48 fractions. Also, considering that the chromatographic setup is crucial for the MS analysis, we tested a 2 h gradient *versus* a 3 h gradient, concatenating two fractions, and a 3 h gradient, concatenating four fractions. The results (not shown) supported the decision to proceed using a 3 h gradient and concatenating 2 fractions. Once the strategy was set, we proceeded with the LC-MS/MS analysis of kidney samples of mice after 3, 6, and 24 h of venom or venom + antivenom injection.

In order to measure the efficiency of TMT labeling, the metrics related to different variable modifications on key residues, especially TMT labeling on Lys at the C-terminus of peptides, was calculated (Supplemental Fig. S1*A*), resulting in a labeling efficiency >95%. Considering that the efficiency of trypsin cleavage should decrease with tagging Lys residues with TMT, it was not expected to detect more than 5% of peptides containing C-terminal Lys residues, while a high percentage of peptides containing free Lys residues at C-terminus would indicate that trypsin was able to cleave internal peptide bonds containing untagged Lys residues. Additionally, the numbers of TMT-tagged N-termini were acceptable for analysis as evidenced by using Lys (+304.2071) as a variable modification in the search in FragPipe (Supplemental Fig. S1*B*). Further quality control applied to the analysis of TMT-labeled peptides is the relative reporter ion signal for each channel separately.

**Fig. 3:**
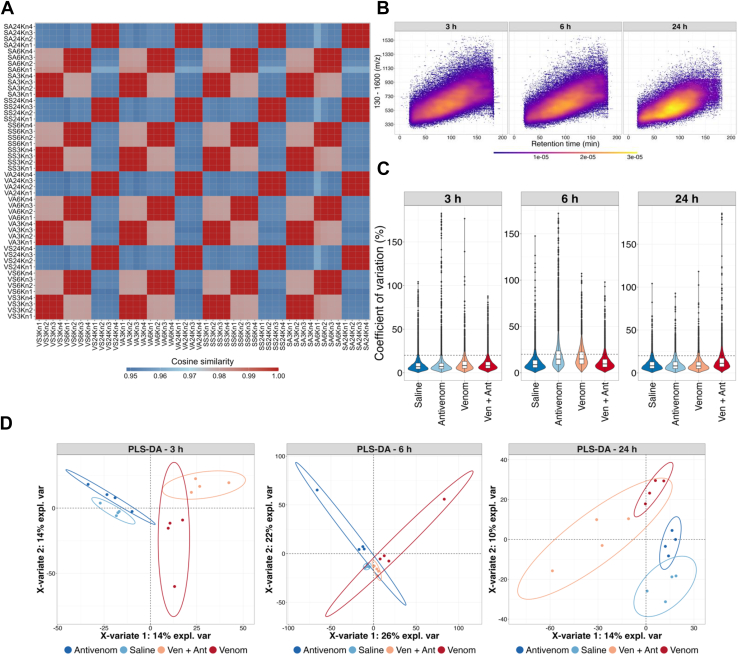
**Analysis of TMT-labeled peptides of kidney tissue samples.***A*, cosine similarity matrix of TMT ion reporters depicting agreement between paired individual animal samples. *B*, ion map extracted from PSM of each TMT-mix. Y-axis: scan range used to measure ions (130–1600 m/z). X-axis: retention time in minutes showing the 3 h gradient. Color gradient is density of the ions measured at each m/z per retention time. *C*, coefficients of variation for each condition and time point. *Dashed line* is 20%. *D*, partial least square discriminant analysis (PLS-DA) of differential protein abundance in kidney samples. *Ellipses* represent the 95% confidence intervals.

The cosine similarity matrix of TMT ion reporters for each analyzed sample revealed consistency between the replicates (Fig. 3*A*). Furthermore, the wide scan range used (130–1600 m/z) resulted in the detection of many ions (Fig. 3*B*). In total, we were able to identify 7506 unique proteins in the three TMT-mixes corresponding to the groups of animals euthanized after 3 h, 6 h and 24 h (Supplemental Table S1). The coefficients of variation for protein abundance in biological replicates among the TMT-mixes were below 20% indicating good precision and low variation between replicates (Fig. 3*C*).

Considering the pronounced local effects of the *Bothrops* snakebite envenomation and the stress induced by the disturbances in hemostasis and cardiovascular system, animals injected with venom alone were expected to show different profiles of kidney protein abundance compared to animals which received only saline or antivenom. Indeed, a partial least square-discriminant analysis (PLS-DA) of the protein abundance data showed a good distinction of animals that were injected saline, venom, antivenom, or venom + antivenom as can be observed by the well-separated experimental groups (Fig. 3*D*).

### Acute-Phase Proteins and Kidney Injury Markers Show Increased Abundance After Venom Injection in the Thigh Muscle

**Fig. 4:**
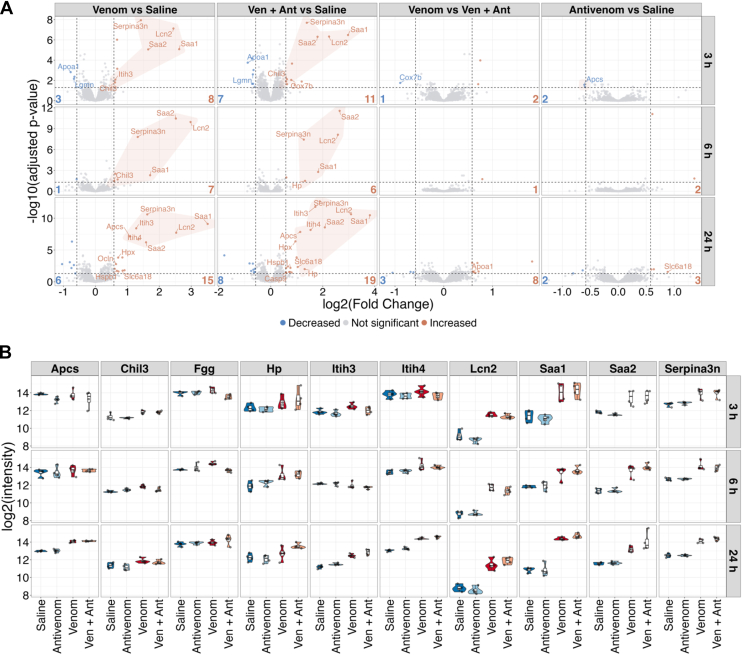
**TMT-based proteomic relative quantification of kidney tissue proteins.***A*, Volcano plots depicting statistically differentially abundant proteins in the kidney of mice injected with *B. jararaca* venom (venom *versus* saline), or with *B. jararaca* venom followed by antivenom (ven + ant *versus*saline), or with antivenom (antivenom *versus* saline) analyzed after 3 h, 6 h, and 24 h, with a limma-based statistical analysis and batch correction. *Dashed horizontal lines* delimit the cut-off for significance considering the adjusted *p*-value ≤ 0.05. *Dashed vertical lines* show the minimum effect size considered relevant as log2FC ≥ |0.58|. The shaded areas indicate inflammation markers. *B*, Violin plots of differential abundance of 10 inflammatory markers identified as increased in mice injected with venom and venom + antivenom (serum amyloid P component, Apcs; chitinase-like protein 3, Chil3; fibrinogen gamma chain, Fgg; haptoglobin, Hp; inter-alpha-trypsin inhibitor heavy chain 3 and 4, Itih3 and Itih4; neutrophil gelatinase-associated lipocalin, Lcn2; serum amyloid A1 and A2, Saa1 and Saa2; and serine protease inhibitor A3N, Serpina3).

A linear model applied to the proteomic data allowed to detect a small, but relevant number of differentially abundant proteins in the mouse kidney samples after venom injection in gastrocnemius muscle. The analysis revealed acute phase markers as the main proteins differentially regulated in abundance. Regardless of the time passed from the venom injection (3, 6 or 24 h), the inflammatory markers remained increased (Supplemental Table S1; Fig. 4). Notably, at the dose used in this study, the antivenom administration was not able to alter the profile of increased acute phase proteins detected in kidneys.

In the group of mice not treated with antivenom, significant differential abundance (*p*_adjusted_ < 0.05) of 8, 7, and 15 proteins, identified as increased after, respectively, 3 h, 6 h, and 24 h of venom injection. In mice that received antivenom 1 h after venom injection, 11, 6, and 19 proteins were identified as differentially increased in abundance after 3 h, 6 h, and 24 h, respectively. The numbers of proteins significantly decreased in abundance were rather lower in mice that received only venom injection, after 3 h, 6 h, and 24 h (Fig. 4*A*). These results also suggested that the impact of the antivenom alone in the kidneys is small in terms of proteome changes, highlighting the well-known safety profile of the antivenom containing (Fab')_2_ heterologous immunoglobulins. Consistent with the kidney pathology occurring in *Bothrops* human envenomation, the injection of *B. jararaca* venom caused consistent increase in abundance of acute-phase proteins (serum amyloid A-1 and A-2, Saa1 and Saa2; serum amyloid P-component, Apcs; fibrinogen gamma chain, Fgg; inter alpha-trypsin inhibitor, heavy chain 3 and 4, Itih3 and Itih4) and of the kidney injury markers serine protease inhibitor A3N (Serpina3n), chitinase-like protein 3 (Chil3), and neutrophil gelatinase associated lipocalin (Lcn2), with lipocalin 2 and serum amyloid A showing the highest change in all the comparisons (Fig. 4*B*) (52, 53, 54, 55). In summary, the differential analysis showed canonical acute phase proteins and lipocalin, a hallmark of renal injury, in higher abundance in the kidneys of animals injected into the muscle with *B. jararaca* venom, from 3 h up to 24 h after the venom injection, regardless of the administration of the pentavalent antivenom.

### Proteolysis Measured in Kidney Samples Indicates the Participation of Endogenous Proteases in the Pathophysiology of Envenomation

To evaluate the role of proteolysis in the kidney response to *B. jararaca* venom injection in the thigh muscle, we investigated the tissue degradome by filtering the PSMs with the highest purity and tagged with TMT at the N-terminus, then normalized them by protein abundance measured from specific peptides using Fragpipe.

**Fig. 5:**
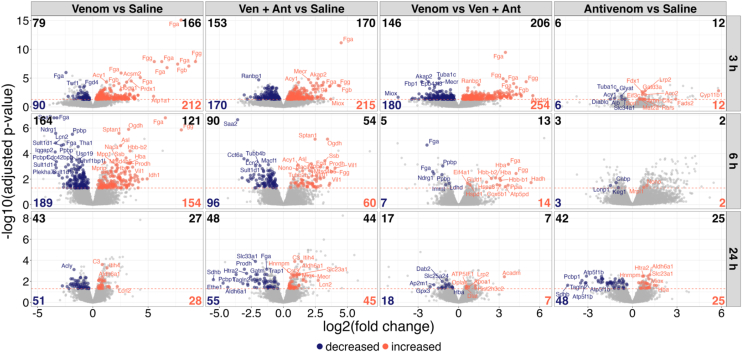
**TMT-based quantitative N-terminomic analysis of kidney tissue proteins.** Volcano plots depicting statistically differentially abundant N-terminal peptides in the kidney of mice injected with *B. jararaca* venom (venom *versus* saline), or with *B. jararaca* venom followed by antivenom (ven + ant *versus* saline), or with antivenom (antivenom *versus* saline) analyzed after 3 h, 6 h, and 24 h, with a limma-based statistical analysis and batch correction. *Gray dots*: peptides with non-significant abundance variation according to the feature specific adjusted *p*-value. Numbers of proteins target of proteolysis extracted from the differentially abundant peptides with neo N-termini per group of comparison and time point are shown in *black* on the *top* of each *panel*.

As expected, animals which received only antivenom had no evidence of significantly affected proteolysis detected in the kidney tissue (Fig. 5). On the other hand, 212, 154, and 28 peptides were identified as significantly increased because of venom injection in the thigh muscle after 3, 6, or 24 h, respectively. These peptides derived from 166, 121, and 27 proteins, respectively (Supplemental Table S2; Fig. 5). In animals injected with venom + antivenom the numbers of proteolytic products and cleaved proteins were similar to those of animals that only received venom: 215, 60, and 45 neo-termini significantly increased were detected after 3 h, 6 h, or 24 h of venom injection. These peptides were derived from 170, 54, and 44 proteins, respectively (Fig. 5).

**Fig. 6:**
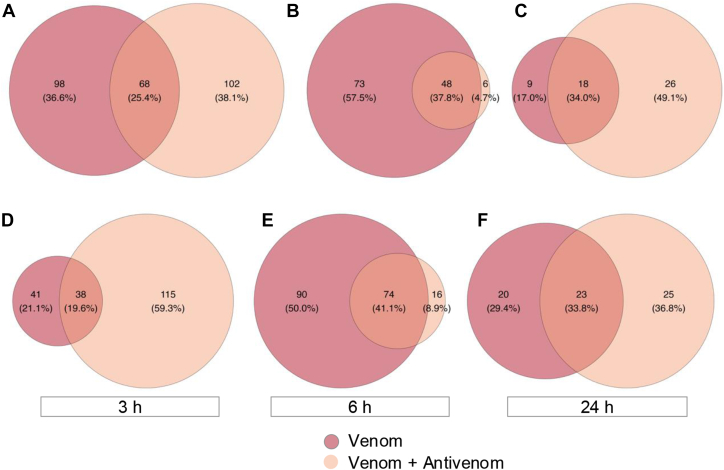
**Comparison of endogenously truncated proteins and peptides in the kidney tissue.***A–C*, Venn diagrams depicting the numbers of truncated proteins as identified by neo-N-terminal peptides significantly increased in the comparison of mice that received venom or venom + antivenom. *D–F*, Venn diagrams depicting the numbers of peptides further processed as identified by neo-N-terminal peptides significantly increased in the comparison of mice that received venom or venom + antivenom.

Although the numbers of differentially abundant peptides were close, their origin was intriguingly distinct. From 268 proteins identified as degraded in mice injected with venom and administered or not with antivenom, only 25.4% (68 proteins) were common between the groups at the 3 h time point (Fig. 6*A*). After 6 h of venom injection, the targets of proteolysis were mainly identified in the group that did not receive antivenom injection, while only six proteins were detected as degraded exclusively in mice injected with venom and antivenom (Fig. 6*B*). However, after 24 h proteolysis dramatically decreased in animals injected only with venom or venom + antivenom, and only 53 proteins degraded at this time point and 49.1% were detected only in mice that received antivenom (Fig. 6*C*). In addition, the analysis of further processed peptides, 59.3% of the targets were in the group of mice that received the venom + antivenom (Fig. 6*D*), while only 19.6% of the peptides were common between the two groups. On the other, 6 h after venom injection 50% of the further processed peptides were detected in the mice injected only with venom, while 41.1% of the peptides were shared with the group injected with venom + antivenom (Fig. 6*E*). After 24 h, the numbers of further processed peptides were similar between the two groups (Fig. 6*F*).

We further evaluated the biological processes that could be disrupted by proteolysis in the kidney tissue. Using the overrepresentation analysis for the proteins from which proteolytic products were identified 3 h after venom injection, we found that only six biological processes were disrupted in mice that received venom + antivenom in comparison to 15 biological processes in those that received only venom, indicating a positive effect of the antivenom (Supplemental Fig. S2).

**Fig. 7:**
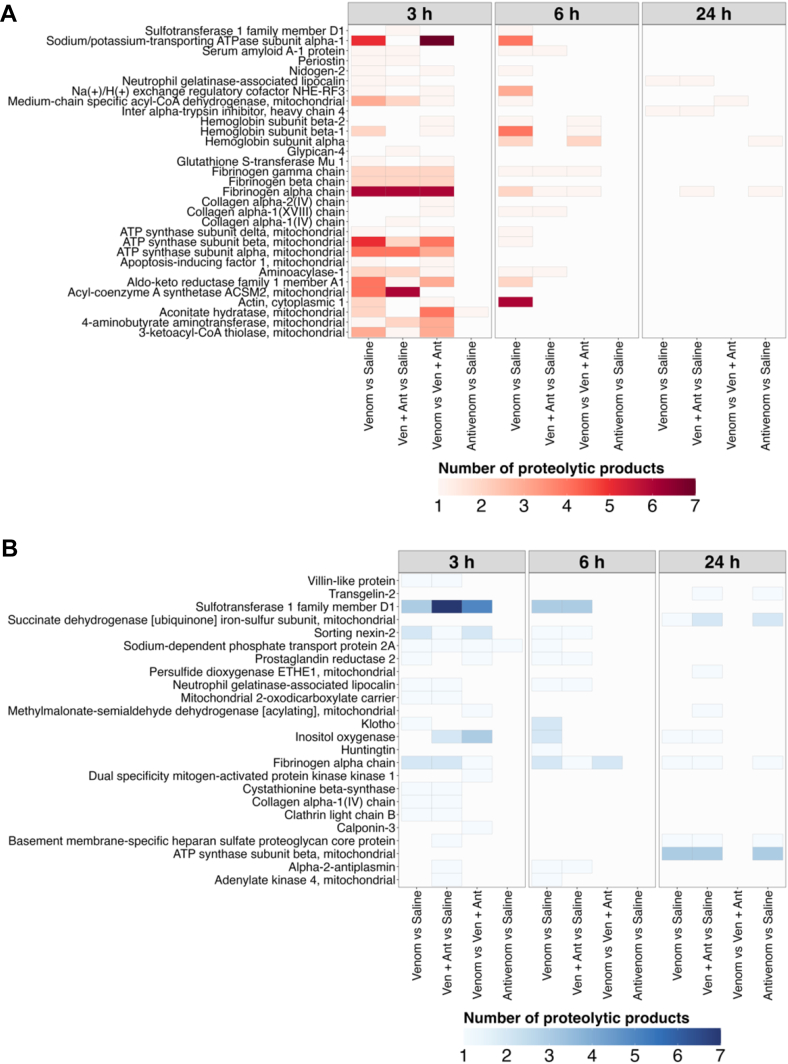
**Main targets of proteolysis as identified from peptides differentially abundant in different comparison groups.***A*, proteins cleaved (Y-axis) on each comparison (X-axis). The color gradient is proportional to the number of peptides found significantly increased for the protein at each comparison. *B*, peptides further processed (Y-axis) on each comparison (X-axis). The color gradient is proportional to the number of peptides found significantly decreased for the protein at each comparison.

Proteolysis in the kidneys of mice injected with *B. jararaca* venom is a dynamic process where peptides coming from different fibrinogen chains were cleaved after 3 h and further degraded after 6 h (Fig. 7). After 24 h, however, the cleavage of fibrinogen and other acute phase proteins decreased (Fig. 7*A*). Interestingly, the antivenom was able to attenuate the cleavage of fibrinogen chains in animals after 6 and 24 h of venom injection. The proteins more proteolytically degraded in our analysis were fibrinogen, hemoglobin, several mitochondrial proteins, and two important proteins to maintain the body-fluid homeostasis (Na+/K+ transporting ATPase subunit alpha 1 and Na(+)/H(+) exchange regulatory cofactor NHE-RF3), whose loss is also involved in kidney injury (56, 57). Moreover, it was possible to detect several peptides from fibrinogen alpha chain, sorting nexin-2, and a sulfotransferase D1 being degraded after venom injection across the time (Fig. 7*B*).

**Fig. 8:**
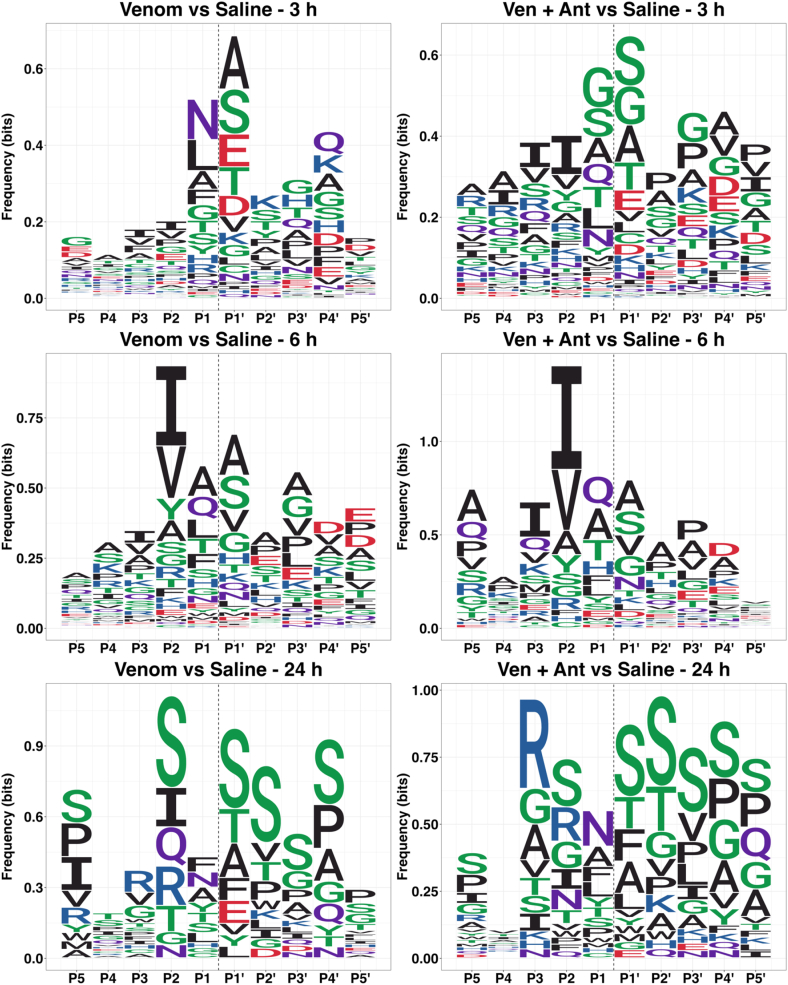
**Proteomic identification of protease cleavage sites (PICS).** Sequence logos depicting the occurrence (in %) of each amino acid residue at P5-P5′ positions in peptides identified in higher abundance in the kidney tissue after 3 h, 6 h and 24 h of *B. jararaca* venom or venom + antivenom injection in the thigh muscle. Peptides up-regulated in PICS analysis represent proteolytic products enriched in each condition tested.

The analysis of protease specificity using the Proteomic Identification of protease Cleavage Sites (PICS) methodology, considering the differentially increased peptides resulted in identification of Asn and Leu as the most frequent residues at P1 position, and Ala and Ser at P1′, after 3 h of venom injection, and indicated that the antivenom altered the proteolysis profile, with Gly and Ser as predominant at the P1 and P1′positions (Fig. 8). According to the MEROPS database, the proteolytic signature identified in the kidney tissue of animals injected only with venom is indicative of the activity of cathepsins or legumain, suggesting that during the process of envenomation, endogenous proteases might play a role in the kidney pathophysiology. In addition, the preference for aliphatic residues (Ile and Val) at the P2 position also indicates the activity of cathepsins (58). Interestingly, the proteolysis profiles at the 6 h and 24 h time points were distinct from those of 3 h, regardless of the presence of antivenom, indicating a change in the preference of amino acid residues at the cleavable peptide bond, however, keeping the possible signature of cathepsins. Overall, these results indicate that Arg/Lys and Leu were not the preferred amino acids at, respectively, the P1 and P1′ positions, which are the primary specificity of SVSPs and SVMPs, respectively (30, 59, 60, 61).

**Fig. 9:**
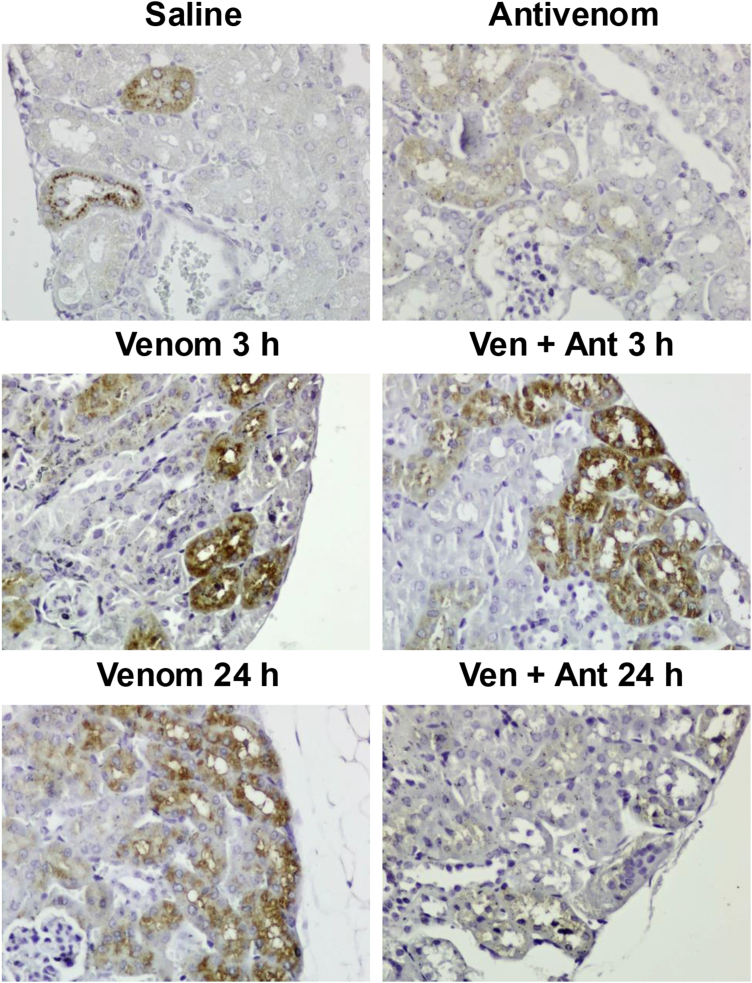
**Immunohistochemical analysis of kidney tissue with an anti-legumain antibody.** FFPE samples were probed with anti-legumain antibody and counter-stained with hematoxylin. *Brown areas* are positive for the presence of legumain.

To further explore the participation of endogenous proteases in the degradation of proteins, we probed the kidney tissue with an anti-legumain antibody by immunohistochemical analysis. As a result, the well-defined staining of legumain was observed in the samples of animals injected with venom and venom + antivenom, at the 3 h time point, in comparison to a basal level of expression in control animals (saline or antivenom). As for the 24 h-samples, however, only those from animals injected with venom showed the clear presence of legumain, while those from animals also administered with antivenom showed no staining (Fig. 9).

### The Mouse Natural Kidney Tissue N-Terminome

The results of the N-terminomic analysis were also used to profile the natural kidney degradome, i.e., the set of small peptides or fragments derived from the truncation of proteins occurring naturally in the tissue as a result of basal proteolysis. For that, a non-specific search (no-enzyme) was conducted in FragPipe, independent of the previous analyses, to select PSMs containing a TMT-tag at the N-terminus and not Arg or Lys as previous residue (semi-N). The correspondent peptides were further filtered by variance in log2(abundance) and only those showing near zero variance were kept (variance <0.05), meaning they were consistently present in the tissue. This set of peptides comprised 623 unique peptides containing Arg or Lys at the C-terminus, or non-semi-C (from truncated proteins), and 70 unique peptides not matching trypsin specificity at the C-terminus, or semi-C (peptidome), composed mostly of 7 to 15 amino acids (Supplemental Table S3; Supplemental Fig. S3*A*).

Interestingly, although these peptides have not been generated by the venom injection in the animals, the sequence logos resulting from the PICS analysis showed Asn as preferred amino acid at the P1 position pointing to the activity of legumain (Supplemental Fig. S3*B*). These peptides compose the basal degradome, with products derived from the cleavage for processing, activation, post-translational modification, or recycling of more than 500 different proteins that play roles in different cell metabolism pathways, present in diverse cell compartments and exerting molecular functions mainly related to energy and respiration (Supplemental Fig. S3*C*).

Next, we explored the relationship between mRNA expression/abundance and protein levels to examine how this relationship compares with basal protein processing/truncation. For that we compared the mRNA levels measured in healthy mice in a previous study (62), and the levels of proteins that were detected as constitutively cleaved/degraded in the present study. Supplemental Fig. S4*A* shows a plot of both z-scored mRNA abundance *versus* protein abundance where the quadrant corresponding to high abundant mRNA and low abundant protein contains most datapoints, revealing a scenario of active mRNA generation for proteins undergoing turnover. These proteins play mainly roles in cell energy and metabolism, and protein synthesis (Supplemental Fig. S4*B*).

### Phosphoproteomic Analysis Provides New Perspectives to Understand the Pathophysiology of Envenomation in the Kidneys

**Fig. 10:**
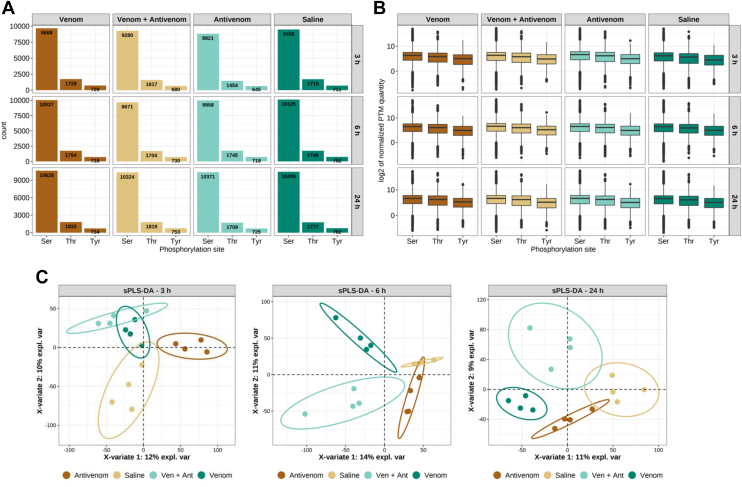
**Phosphoproteomic analysis of kidney samples.***A*, numbers of unique phosphorylation sites identified in serine, threonine, and tyrosine residues in each experimental group and per time point. *B*, box plots depicting phosphopeptide abundance normalized by protein abundance. *C*, sparse partial least-squares discriminant analysis of phosphopeptides performed by time point using the samples as response variable. *Ellipses* represent the 95% confidence intervals.

Phosphorylation is the most frequent, reversible post-translational modification (PTM) in eukaryotic proteins, which regulates many protein features, such as function, subcellular localization, degradation, complex formation, and activation, thereby directly influencing cell signaling networks of biological processes in health and disease. In this study we evaluated for the first time the participation of altered phosphorylation in the venom-induced kidney pathology. We mapped 22,774 unique phosphosites in mouse proteins of all experimental groups. The numbers of unique phosphorylation sites found in each experimental condition was consistent with the residue distribution as expected for eukaryotic organisms, i.e. high frequency of phosphorylated serine residues, followed by threonine and tyrosine residues (Fig. 10*A*) (63). Following the distribution of amino acids identified as phosphorylated, the peptide abundances were consistent (Fig. 10*B*). After filtering for protein groups q-value <0.01 and PTM site probability >0.95, a normalization of the phosphopeptide abundances by their respective protein abundances was applied, allowing for an evaluation of the fraction of peptide abundance not related to protein abundance. Overall, the identification of high confident phosphorylation sites was higher than 10,000 per sample (Fig. 10*A*).

Furthermore, the values of phosphopeptide abundance normalized by protein quantity were used to discriminate the samples using a sparse partial least-squares discriminant analysis across the different experimental time points resulting in the clear discrimination between the mice injected only with saline or antivenom (controls) and those injected with venom or venom + antivenom (Fig. 10*C*). Hence, this indicated that phosphorylation cascades in the kidney tissue were perturbed by the venom injection as early as 3 h and up to 24 h after venom injection in gastrocnemius muscle.

Considering the power to discriminate between the experimental groups (Fig. 10*C*), the linear model applied to phosphopeptide identification after normalization by the protein abundance indicated that signaling events controlled by phosphorylation in the kidneys was remarkably altered 3 h after venom injection, regardless of antivenom administration. Notably, specifically after 24 h, the groups of animals that received saline or antivenom clearly separated, indicating a particular effect of the antivenom itself in perturbing the phosphorylation signaling in the kidneys. Nevertheless, the comparison of phosphorylation in mice injected with venom and venom + antivenom is suggestive that the antivenom had an effect on the phosphorylation profile of the kidney tissue only at the 3 h time point.

**Fig. 11:**
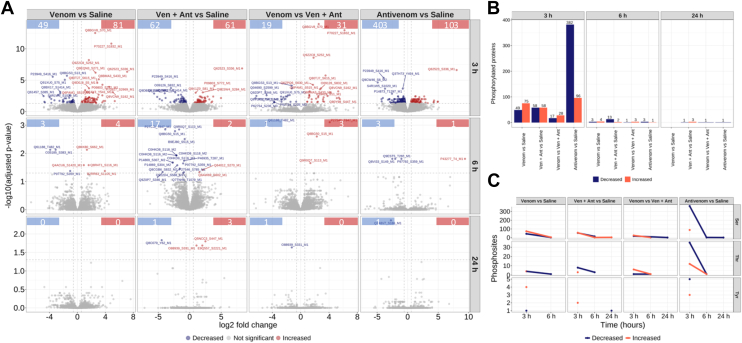
**Phosphoproteomic analysis of kidney tissue.***A*, differentially abundant phosphopeptides in comparisons produced in limma analysis after peptide abundance normalization by protein abundance. Phosphopeptide abundance was considered differentially regulated when adjusted *p*-value ≤ 0.05. *Dashed lines* indicate adjusted *p*-value cut-off (*horizontal*) and log2 of fold change ≥ |0.58|. *Boxes* indicate the number of phosphopeptides found differentially increased (*red*) or decreased (*blue*). *Boxes* show the numbers of differentially regulated phosphopeptides. *B*, numbers of proteins with phosphorylation events differentially regulated in each condition and time point. *C*, numbers of differentially regulated phosphorylated residues per comparison across the time.

In all cases, the numbers of phospho-regulated sites in the kidney tissue were decreased 24 h after venom injection (Supplemental Table S4; Fig. 11). Indeed, the investigation of proteins with differentially phosphorylated sites revealed higher numbers at the 3 h time point in all experimental groups, except for the animals that only received saline, which however dramatically decreased after 6 h (Fig. 11*A*), and the profile of amino acid residues differentially phosphorylated showed predominantly serine (Fig. 11*B*). The number of increased (103) or decreased (403) phosphorylation sites in mice that injected only with antivenom was intriguingly higher in comparison to the mice injected with venom or venom + antivenom (Fig. 11*A*). The trending of phosphorylation as stratified in experimental group and phosphorylated amino acid residue was characterized by an overall reduction, which was remarkably pronounced in the group of animals injected with antivenom (Fig. 11*C*).

**Fig. 12:**
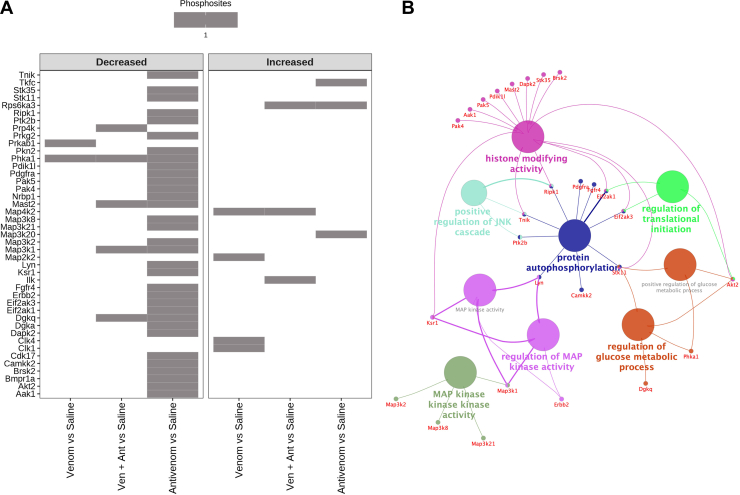
**Differential profile of phosphorylation of 43 kinases in the kidney tissue.***A*, kinases differentially phosphorylated at the 3 h time point in experimental group comparisons produced in limma analysis after peptide abundance normalization by protein abundance. *B*, network of biological processes enriched from the kinases with decreased levels of phosphorylation in the comparison of groups of animals injected with antivenom or saline at the 3 h time point.

The identification of differentially phosphorylated proteins revealed 43 kinases differentially phosphorylated. Unexpectedly, the levels of phosphorylation in kinases from mice that received only antivenom decreased in comparison to other groups, while only the kinases Map3k20 (mitogen-activated protein kinase kinase kinase 20), Rps6ka3 (ribosomal protein S6 kinase alpha-3), and Tkfc (triokinase/FMN cyclase) showed increased levels of phosphorylation in mice that received only antivenom (Fig. 12).

An enrichment analysis of biological processes involving the kinases identified with decreased level of phosphorylation in the kidneys of animals only treated with antivenom revealed nine overrepresented processes involving histone modification, activity, protein autophosphorylation, regulation of glucose metabolic process, regulation of translational initiation, positive regulation of JNK cascade, and regulation of MAP kinase activity (Fig. 12*B*).

Evaluating the pathways affected by the proteins found with phosphorylation events increased, it was possible to find that 17% of the proteins participate in cellular response to toxic substance, cell-substrate junction assembly, pyridine nucleotide metabolic process, 11% participate in actin filament bundle organization and sterol homeostasis (Supplemental Fig. S5). On the other hand, overrepresentation analysis including only proteins with significantly decreased phosphorylation sites showed several biological pathways affected by the antivenom administration (Supplemental Fig. S6). The biological processes most enriched are somitogenesis, carbohydrate homeostasis, intrinsic apoptotic signaling pathway in response to DNA damage, and several terms related to developmental stages.

The Gene Ontology analysis related to the proteins with phosphorylation sites differentially increased after venom injection suggests a dynamic change in biological processes and cellular components moving from apoptotic process and platelet dense tubular network membrane at 3 h, through cellular nitrogen compound biosynthetic process and cell projection at 6 h, to development and sarcoplasmic reticulum at 24 h (not shown). In mice injected with venom and treated with antivenom the main biological processed and cellular components enriched were regulation of protein depolymerization and microvillus at 3 h, and positive regulation of cellular component organization and apical junction complex at 6 h. At 24 h there was no enrichment given the low number of proteins found with regulated phosphorylation sites.

## Discussion

*Bothrops* venoms are recognized for eliciting local effects, generating local hemorrhage and inflammatory response, as well as systemic effects, including coagulopathy, in human envenomation and by injection in different animal models. Therefore, a comprehensive profiling of the venom pathogenesis is only possible by the application of system-wide analyses of toxin targets by omics methods. Here we explored the mammalian kidney response to *Bothrops* envenoming in a mouse model, by the intramuscular injection of *B. jararaca* venom in the thigh muscle, mimicking a snakebite, followed by the intravenous injection of anti-*Bothrops* antivenom. The venom induced clear alterations in the kidneys, which were detected at distinct levels of complexity, including proteome, degradome and phosphoproteome.

Although there were no remarkable morphological changes in the kidney tissue of mice injected with saline or with venom or with venom + antivenom up to 24 h (Fig. 2), it was possible to observe alterations in part of the red blood cells that showed transient acanthocytosis, which spontaneously resolved regardless of antivenom administration. Acanthocytes may develop from anomalies in membrane lipids and proteins (64). In animals injected with venom, these morphological alterations in red blood cells may be related to the activity of venom phospholipases A2, although the participation of metalloproteases in this process cannot be ruled out (65, 66). Important tissue morphological alterations have been reported in kidneys perfused with 3 μg/ml of *B. alternatus* venom in an isolated kidney rat model (67). Also using a rat model, the intramuscular injection of *B. jararaca* venom up to 8 mg/kg resulted in changes in kidney biochemical parameters and glomerular injury with collagen deposition (68). While these and other similar studies have provided useful insights on the envenomation process, the animal models employed are not comparable to our study. As snakebite-related AKI is a common complication following envenomation by snakes of the *Bothrops g*enus, our results indicate that possibly, the histopathological signs of kidney injury would take more than 24 h to become evident in the mouse kidney or require a higher dose of venom to cause alterations earlier than 24 h. Here, the venom dose injected (1.6 mg/kg animal body weight) was slightly below the LD_50_ of the Brazilian Bothropic Reference Venom (1.79 mg venom/kg animal body weight) (69) and we followed the mice up to 24 h.

As different types of protein N-termini may be present in the protein extract of mammalian samples, depending on the activity of proteases, and other post-translational modifications, we applied a TMT-labeling workflow combined with semi-specific search of MS spectra to achieve accurate information on endogenous protease cleavage and global proteomic analysis with exceptional depth (47). This approach proved to be efficient and effective for extracting meaningful information on proteolytic events from large-scale explorative proteomics data, with the identification of over 7500 unique proteins and 7000 semi-specific and/or N-terminally TMT-tagged peptides in three TMT-mixes composed of samples from the groups of animals euthanized after 3, 6 and 24 h of the first injection. Notably, this strategy outperformed previous similar analyses, using a Q-Exactive Plus mass spectrometer, of the kidney tissue proteome in the context of polycystic kidney disease in a mouse model, which resulted in the identification of 5379 proteins and 3005 semi-specific and/or N-terminally TMT-tagged peptides (47). These results emphasize the advantage of decreasing the complexity of peptide samples in offline pre-fractionation using high-pH reversed-phase chromatography and choosing a long elution gradient in LC-MS/MS to increase proteome and N-terminome coverage.

Although the number of proteins with increased abundance in the kidney tissue was low (8, 7, and 15 proteins identified after, respectively, 3, 6, and 24 h of venom injection), in mice that were not administered with antivenom, the alteration in inflammatory markers could be detected as soon as 3 h and remained significantly increased after 24 h (Fig. 4). Besides, the antivenom administration was not capable of attenuating the inflammatory status of the kidney proteome in this animal model. AKI is characterized by the interplay of damaging interactions between the tissue microvasculature, tubular epithelial cells, and infiltrating inflammatory cells. Acute-phase proteins are mainly produced in the liver and secreted to the circulation, from where they can reach virtually any system in the body. In response to tissue injury or infection, activated macrophages secrete cytokines that induce liver synthesis of a number of acute phase reactants (70, 71). Among the acute-phase proteins detected in higher abundance in animals injected with venom three types of serum amyloid proteins (Saa1 and Saa2, and Apcs) are highly conserved proteins that play a role in acute and chronical inflammation. Saa1 and Saa2 are known to interact with several receptors such as Toll-like receptors, formyl peptide receptor 2, scavenger receptor class B type I, ATP receptor P2X7, and to activate NF-κB triggering a pro-inflammatory response (72), thus contributing to the damage of envenomation. Apcs is a pentraxin member of the humoral arm of the innate immune system known to activate complement and to promote phagocytosis of cell debris in response to tissue damage (73, 74). Also detected in higher abundance were the inter alpha-trypsin inhibitors 3 and 4 (Itih3 and Itih4). These are highly abundant proteins in mammalian serum, containing two heavy chains and one light chain. While the light chain (bikunin) is responsible for protease inhibitory activity, the heavy chains have the capacity to bind to hyaluronan *via* a transesterification reaction causing aggregation of the complex protein/glycosaminoglycan and contributing to the deposition of new extracellular matrix (75). Interestingly, the heavy chains can also play a role in the negative feedback of the early complement system activation by inhibiting complement activation locally in the tissue (76). Hence, while Apcs can activate complement and impose some risk of injury to the kidney parenchyma, Itih3 and Itih4 might balance the detrimental effect protecting the tissue from the complement attack complex. The glycoprotein chitinase-like protein 3 (Chil3) is strongly expressed by macrophages in inflammatory diseases. The normal abundance of this protein in plasma is unknown, but its increase is well documented to correlate with several inflammatory diseases, such as cardiovascular disease, neurological diseases, cancer, and autoimmune diseases (77). The fibrinogen gamma chain (Fgg) plays physiological roles in fibrin formation, platelet aggregation, and wound healing, but its increase in abundance, as detected here in the mouse tissue, may contribute to inflammation. The binding of fibrinogen to leukocyte alphaMbeta2 integrin is mediated by different epitopes and is involved in the recruitment of phagocytes during inflammation (78, 79, 80). Also, it has been shown that the transglutamination of the gamma chain by factor XIIIa generates clots more resistant to fibrinolysis, which would contribute to fibrin deposition in the kidney tissue. As, in principle, all forms of tissue damage, irrespective of the promoting agent, involve the local activation of the blood clotting system, it is expected that the higher level of Fgg plays a role in the kidney response to the venom insult.

Likewise, among the proteins detected in higher abundance, lipocalin 2 (Lcn2) or neutrophil gelatinase-associated lipocalin (NGAL) and the serine protease inhibitor A3N (Serpina3n), are related to the progress of kidney diseases (52, 55). Lcn2 has been proposed as biomarker for acute kidney injury based on its quaternary structure. While neutrophils mainly produce the dimeric form of Lcn2, renal tubular cells produce mainly the monomeric form, and both can be detected in urine samples (81, 82). Our analysis does not discriminate between both structural forms of Lcn2 and its increased abundance may have originated from neutrophils or the renal tissue (83). Lcn2 is reported to induce bacteriostasis, and to promote anti-apoptotic effects and an enhanced proliferation of renal tubules, suggesting pathways in which it can act protecting the kidney tissue in acute injury (84). Accordingly, Lcn2 has been reported as a good biomarker in predicting AKI in *Bothrops* human envenoming (85). Serine protease inhibitor A3N (Serpina3n; alpha-1 antichymotrypsin) is a marker of immune response and inflammation in different pathological scenarios. The mechanism underlaying the renal increase of Serpina3n in mice injected with *B. jararaca* venom is unknown but it might occur in response to the protease-promoted degradation of matrix proteins occurring in the tissue. Actually, both Lcn2 and Serpina3n have been detected in higher abundance in the kidneys and blood-plasma of mice injected in the thigh muscle with the venom hemorrhagic metalloprotease HF3 (62).

Overall, the higher abundance of acute-phase proteins indicates venom-induced renal inflammation that might lead to glomerular damage, even if it was not morphologically apparent in our model. Additionally, the fact that globally the kidney tissue proteome remained unchanged up to 24 h after venom injection denotes a robust response to the presence of circulating toxins and to the increased inflammation markers, keeping the organ homeostasis, in the context of the emerging venom-induced nephrotoxicity.

The N-terminomic analysis, on the other hand, underscored the proteolysis scenario occurring in the kidney tissue, with the processing or degradation of a range of proteins, involved in essential biological processes. Neo-N-termini peptides derived from 166, 121, and 27 proteins, were identified after, respectively, 3, 6 and 24 h of venom injection, while animals also treated with antivenom showed a similar profile of proteolysis (Fig. 5). Nevertheless, most of proteins that underwent proteolysis were different in the groups of animals that received only venom or venom + antivenom, indicating some modulation of proteolytic events in the tissue by the antivenom, also reflected in the lower number of biological processes disrupted by proteolysis (Fig. 6). On the other hand, overall, proteolysis measured in the kidneys decreased over time, independentl of the antivenom administration. Proteolytic products generated as a consequence of venom injection could be detected at the kidney tissue after 3 h, with 212 differentially increased neo-terminal peptides, as well as 90 differentially decreased neo-terminal peptides, coming from further processing of natural peptides (Fig. 5).

Proteins degraded in the kidneys include both plasma and tissue components. Notably, peptides derived from the cleavage of the three fibrinogen chains were identified as increased, confirm its targeting by venom-induced proteolysis both in plasma and in kidney tissue (37, 44, 62, 86). Also degraded were the proteins Na+/K+ transporting ATPase subunit alpha 1 (Atp1a1) and Na(+)/H(+) exchange regulatory cofactor NHE-RF3 (Pdzk1). Atp1a1 is a major component of the osmosensory signaling pathway whose deficiency is linked to loss of magnesium ions in the kidney tubules, resulting in seizures and other neurological detrimental effects (87). The protein Pdzk1 is mainly expressed in proximal tubule cells and in addition to sodium control, it is important for the expression and normal function of the Cl-anion exchanger CFEX in the kidneys (88). Yet, the low level of Pdzk1 was reported to predict poor prognostic in chronic renal diseases (89). Interestingly, antivenom administration ameliorated the degradation of the fibrinogen, Atp1a1, and Pdzk1, upon time. Moreover, the degradation of various mitochondrial proteins, especially at the 3 h time point, suggests a perturbation in the generation of ATP with unknown consequences for the kidney tissue (Fig. 7).

N-terminomics allows for the determination of cleavage sites in proteins, providing insights into newly *in vivo*-generated protein fragments, which might play unknown roles in signaling events either synergistically or antagonistically, contributing to pathology. Here, the fingerprinting of proteolytic events occurring in the kidneys is compatible with the activity of lysosomal cathepsins or legumain (Fig. 8). Actually, the high frequency of asparagine and also leucine at the P1 position at the cleavage site of peptides with increased abundance after 3 h of venom injection is highly suggestive of legumain activity, and indeed the immunohistochemical analysis using an anti-legumain antibody showed the higher abundance of the protease up to 24 h (Fig. 9). These findings are intriguingly in disagreement with the proteomics results on legumain abundance showing a significant decrease at 3 h and a slight increase (although not significant) at 6 h and 24 h. We speculate that the reason for this discrepancy could be related to the fact that protein extraction from kidney tissue may not have resulted in a homogeneous (consistent) yield of proteins derived from all cell compartments and extracellular milieu. In the immunohistochemical assay, on the other hand, the cell layers represented in the tissue slice comprise all tissue elements, thus allowing for a more in-depth detection of legumain.

It was remarkable to observe the clear change in the profile of proteolytic events, specifically in the amino acid preference after 6 h of venom treatment, which were clearly different from those at 3 h, and, nevertheless, also point to the activity of cathepsins, even in the animals also treated with antivenom. What is likely causing this profile is the venom protease-promoted activation of host proteases, which ultimately mediate the generation of the bulk of neo-N-terminal fragments and the propagation of damaging effects. Moreover, as a lower number of biological processes were disrupted in animals injected with venom + antivenom, it becomes clear that under the conditions of this study, antivenom administration was effective in the mitigation of the proteolytic detrimental effects of *B. jararaca* venom in the kidneys in the first 3 h after venom injection.

Protein degradation is an essential process of protein homeostasis within cells. Disagreement between the level of mRNA and protein expression has been documented and might be related to differences in transcriptional and translational rates and protein half-life (90). Regarding the kidney tissue, age-related changes in protein that occur in the absence of corresponding changes in mRNA level suggested that some aging processes are not transcriptionally regulated (91). Here we identified the subproteome of proteins that were constitutively degraded, giving rise to the natural N-terminome of the kidney tissue and found that their abundance is in discordance with mRNA level, indicating at any rate a correlation between protein turnover and demand for RNA translation (Supplemental Fig. S4). Furthermore, in parallel to the venom-induced proteolytic events, a background of natural peptides and/or protein fragments, which did not show abundance variation, was concomitantly present in the kidney tissue. In fact, this consisted of a large pool of available molecules that might influence the maintenance of tissue homeostasis disturbed by the venom toxins, suggesting a putative moonlighting role for the natural cell peptidome contributing to the innate immune response. Another insight from the analysis of the N-terminome is that the venom induced changes both in the kidney tissue proteome and peptidome, with consequent homeostatic imbalance illustrated by alterations in various molecular pathways.

Phosphorylation is a critical mechanism in protein activity regulation and cellular signaling in health and disease. Various studies have explored the kidney phosphorylation in different animal models, including a sepsis-induced kidney injury model (92). Current understanding of venom-induced AKI mainly includes alterations in adaptive immune responses to exacerbated inflammation, vasoconstriction leading to renal ischemia, disseminated intravascular coagulation, intravascular hemolysis and coagulation, and glomerular deposition of fibrin (8, 45). However, the global changes of the injured kidneys upon snake envenomation at the phosphoproteome level remained largely unknown. Here, although analyzed by different protocols and equipment, the number of proteins identified by proteomics (∼7500) and phosphoproteomics (∼5100) provided excellent coverage of the kidney tissue. Regarding phosphorylation events, the kidney tissue underwent changes up to 3 h after venom injection, showing 130 altered phosphosites, however with remarkable return to nearly background values after 6 h and up to 24 h, regardless of antivenom administration (Fig. 11). Unexpectedly, the antivenom itself promoted a high number of altered phosphosites and protein targets (506), which nevertheless decreased after 3 h. These events were measured only 2 h after antivenom administration, suggesting the antivenom had some early impact in the kidney phosphoproteome. At least 43 kinases were regulated during the process of response to envenomation and antivenom treatment (Fig. 12). The fact that most differences in the kidney phosphoproteome were detected 3 h after venom injection indicates that phosphorylation is a rapid response from cells to signal alarm due to the perturbation in phospho-homeostasis triggered by the toxins, and unexpectedly, by the antivenom. The direct antivenom effect decreasing the phosphorylation in >30 kinases affected a number of essential signaling processes, which certainly downplayed the tissue response in animals also injected with venom.

The limitation of the interpretation of the results of our study is not related to the depth of our proteomic/N-terminomic/phosphoproteomic data, which indicated excellent coverage in all analytical aspects, but rather to the *in vivo* experimental conditions. Here we used male mice, one dose of venom and of antivenom, and analyzed their effects at three time points (3, 6, and 24 h). Therefore, our interpretation of the outcome of envenomation and impact of antivenom in a mouse model are limited to the applied experimental conditions, bearing in mind that different effects could be detected after 24 h, and if female specimens were included in the study, and if higher doses of venom and antivenom had been used.

Exploring the primary altered pathways associated with renal response to snake envenomation and identifying potential targets from the proteome, degradome, and phosphoproteome sheds light on the direct and indirect mechanisms of toxin activity. This proteomic/degradomic/phosphoproteomic study provides the first molecular landscape of the kidney tissue of mice injected with a viperid snake venom, offering valuable insights for the establishment of envenomation markers and treatment, and pointing out the different levels of molecular response engaged by the organism upon the challenge of snakebite envenomation.

## Data Availability

All mass spectrometry proteomics data have been deposited to Mass Spectrometry Interactive Virtual Environment (MassIVE). Link: ftp://MSV000097066@massive.ucsd.edu.

## Supplemental Data

This article contains supplemental data.

## Conflict of Interest

The authors declare that they have no conflicts of interest with the contents of this article.

## Supplemental Data

Supplemental Table S2

Supplementl Table S3
