# Supplementary material for: Signatures of the Systemic Effects of a Snake Venom and Antivenom: Multiomics Profiling of the Kidney Pathology
Source: Mol Cell Proteomics. 2025 Jun 27;24(8):101023. doi: 10.1016/j.mcpro.2025.101023 (PMC12336009; doi:10.1016/j.mcpro.2025.101023)
Supplement: Supplemental Figs S1–S6 [file mmc1.pptx]

## Slide 1
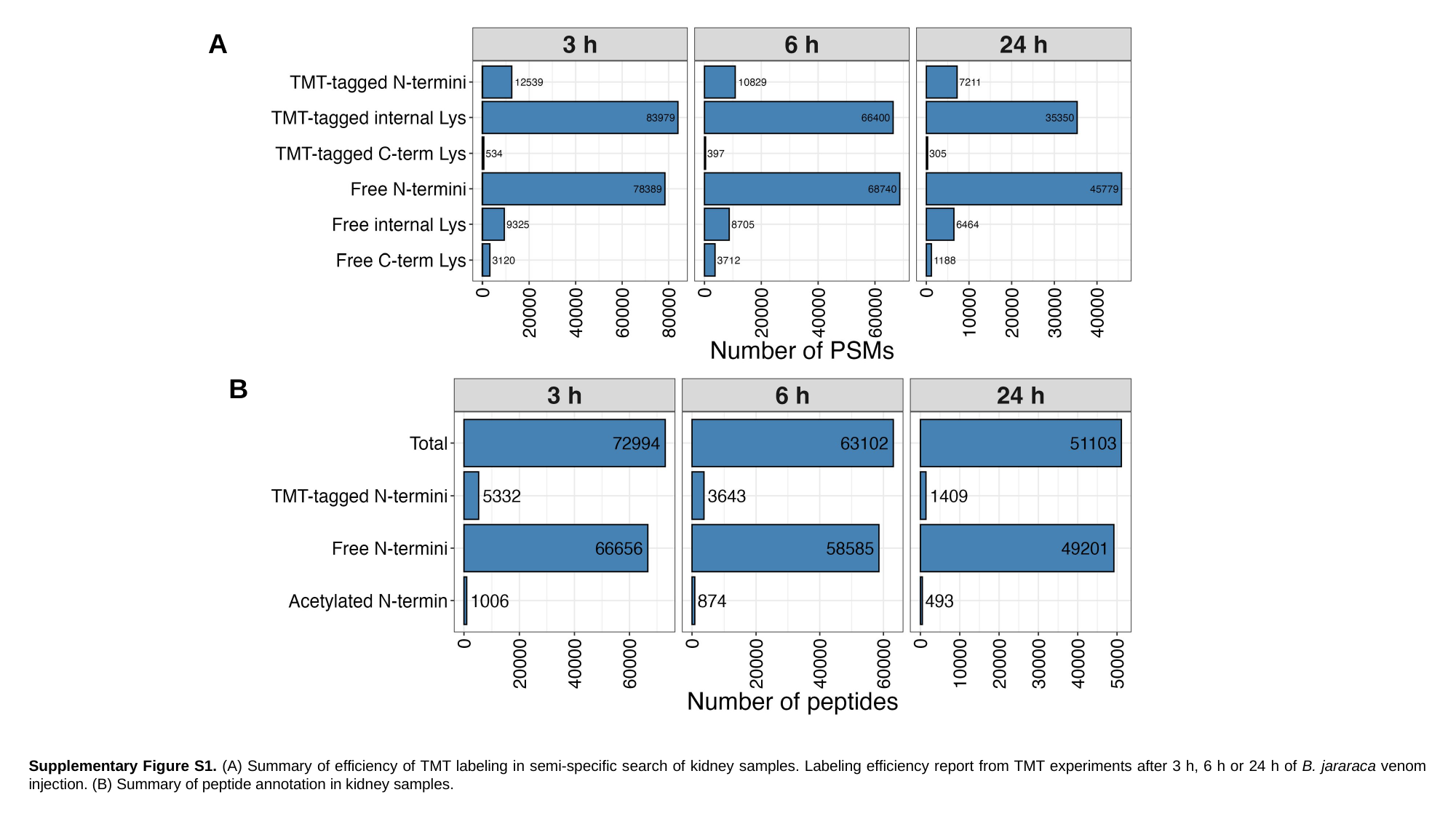

A
B
Supplementary Figure S1. (A) Summary of efficiency of TMT labeling in semi-specific search of kidney samples. Labeling efficiency report from TMT experiments after 3 h, 6 h or 24 h of B. jararaca venom injection. (B) Summary of peptide annotation in kidney samples.

## Slide 2
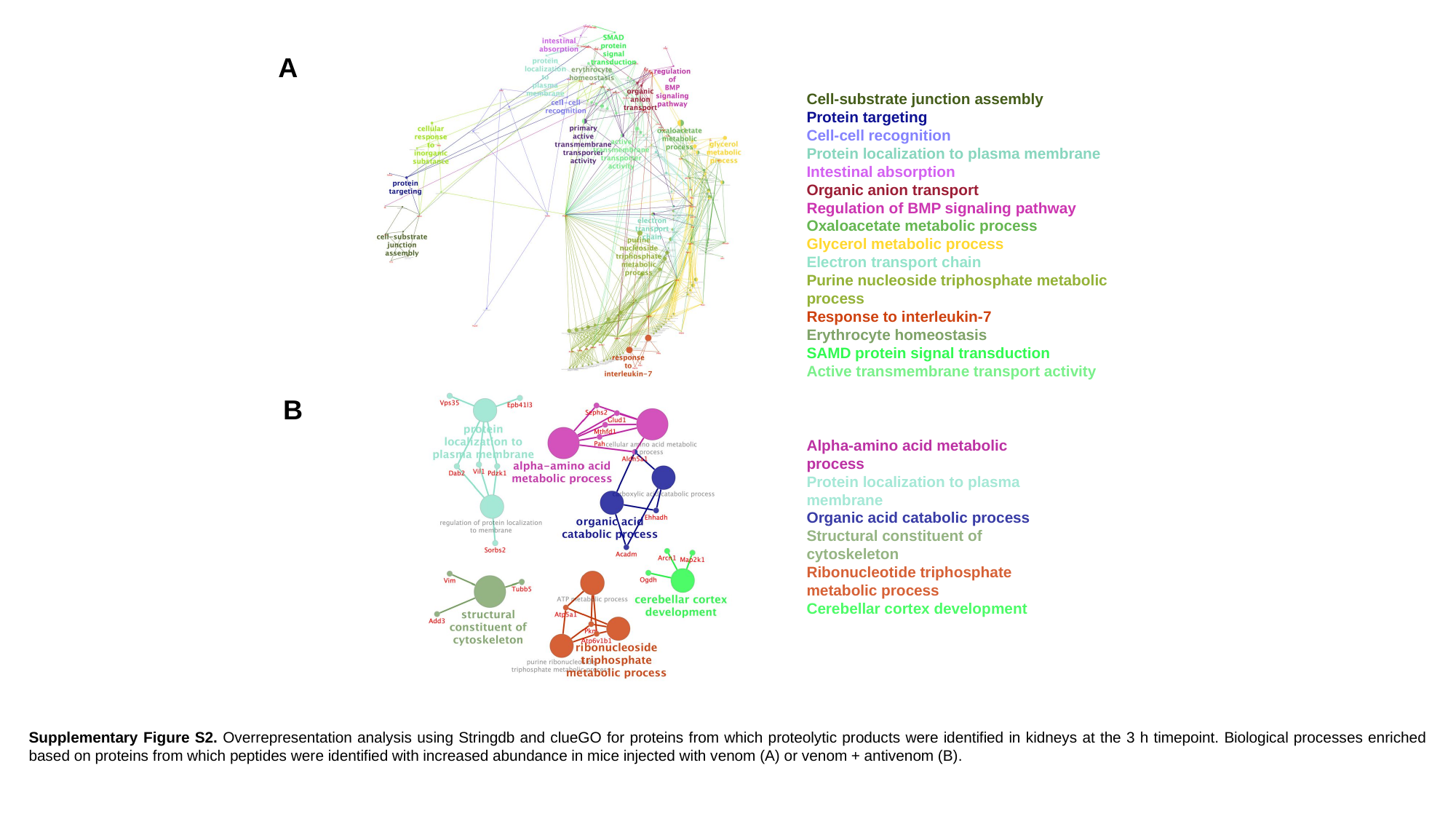

A
Cell-substrate junction assembly
Protein targeting
Cell-cell recognition
Protein localization to plasma membrane
Intestinal absorption
Organic anion transport
Regulation of BMP signaling pathway
Oxaloacetate metabolic process
Glycerol metabolic process
Electron transport chain
Purine nucleoside triphosphate metabolic process
Response to interleukin-7
Erythrocyte homeostasis
SAMD protein signal transduction
Active transmembrane transport activity
B
Alpha-amino acid metabolic process
Protein localization to plasma membrane
Organic acid catabolic process
Structural constituent of cytoskeleton
Ribonucleotide triphosphate metabolic process
Cerebellar cortex development
Supplementary Figure S2. Overrepresentation analysis using Stringdb and clueGO for proteins from which proteolytic products were identified in kidneys at the 3 h timepoint. Biological processes enriched based on proteins from which peptides were identified with increased abundance in mice injected with venom (A) or venom + antivenom (B).

## Slide 3
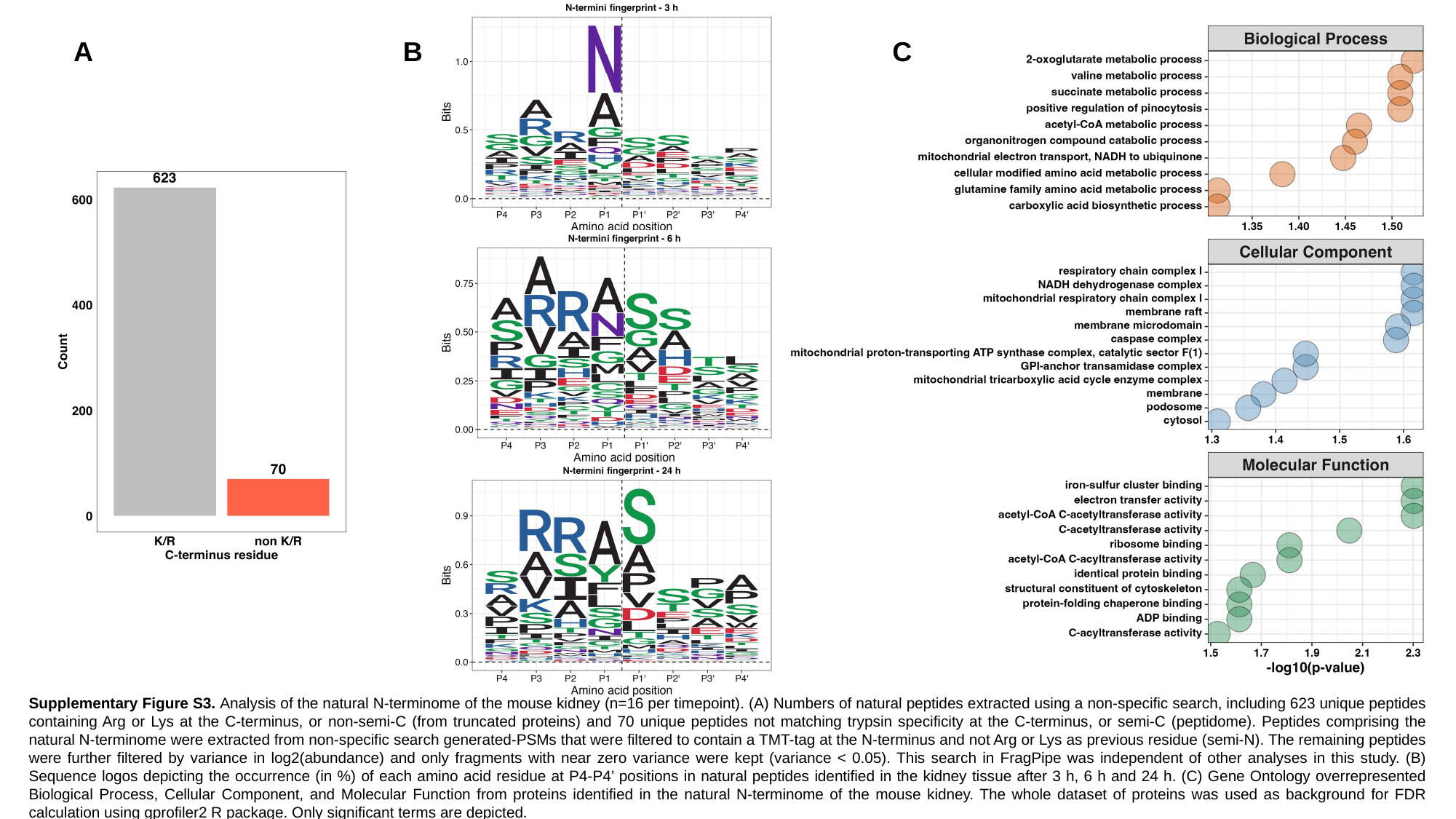

A
B
C
Supplementary Figure S3. Analysis of the natural N-terminome of the mouse kidney (n=16 per timepoint). (A) Numbers of natural peptides extracted using a non-specific search, including 623 unique peptides containing Arg or Lys at the C-terminus, or non-semi-C (from truncated proteins) and 70 unique peptides not matching trypsin specificity at the C-terminus, or semi-C (peptidome). Peptides comprising the natural N-terminome were extracted from non-specific search generated-PSMs that were filtered to contain a TMT-tag at the N-terminus and not Arg or Lys as previous residue (semi-N). The remaining peptides were further filtered by variance in log2(abundance) and only fragments with near zero variance were kept (variance < 0.05). This search in FragPipe was independent of other analyses in this study. (B) Sequence logos depicting the occurrence (in %) of each amino acid residue at P4-P4’ positions in natural peptides identified in the kidney tissue after 3 h, 6 h and 24 h. (C) Gene Ontology overrepresented Biological Process, Cellular Component, and Molecular Function from proteins identified in the natural N-terminome of the mouse kidney. The whole dataset of proteins was used as background for FDR calculation using gprofiler2 R package. Only significant terms are depicted.

## Slide 4
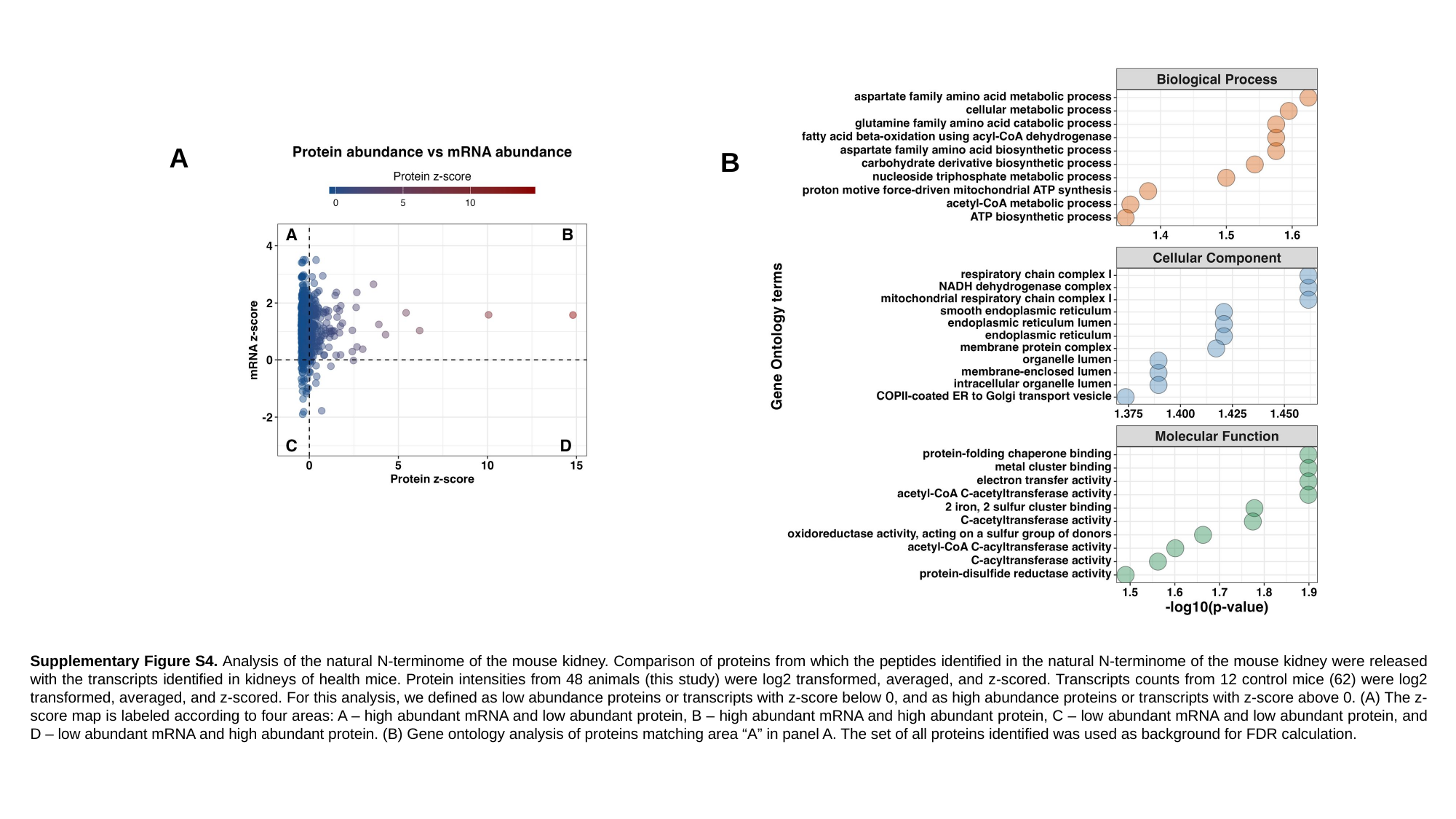

A
B
Supplementary Figure S4. Analysis of the natural N-terminome of the mouse kidney. Comparison of proteins from which the peptides identified in the natural N-terminome of the mouse kidney were released with the transcripts identified in kidneys of health mice. Protein intensities from 48 animals (this study) were log2 transformed, averaged, and z-scored. Transcripts counts from 12 control mice (62) were log2 transformed, averaged, and z-scored. For this analysis, we defined as low abundance proteins or transcripts with z-score below 0, and as high abundance proteins or transcripts with z-score above 0. (A) The z-score map is labeled according to four areas: A – high abundant mRNA and low abundant protein, B – high abundant mRNA and high abundant protein, C – low abundant mRNA and low abundant protein, and D – low abundant mRNA and high abundant protein. (B) Gene ontology analysis of proteins matching area “A” in panel A. The set of all proteins identified was used as background for FDR calculation.

## Slide 5
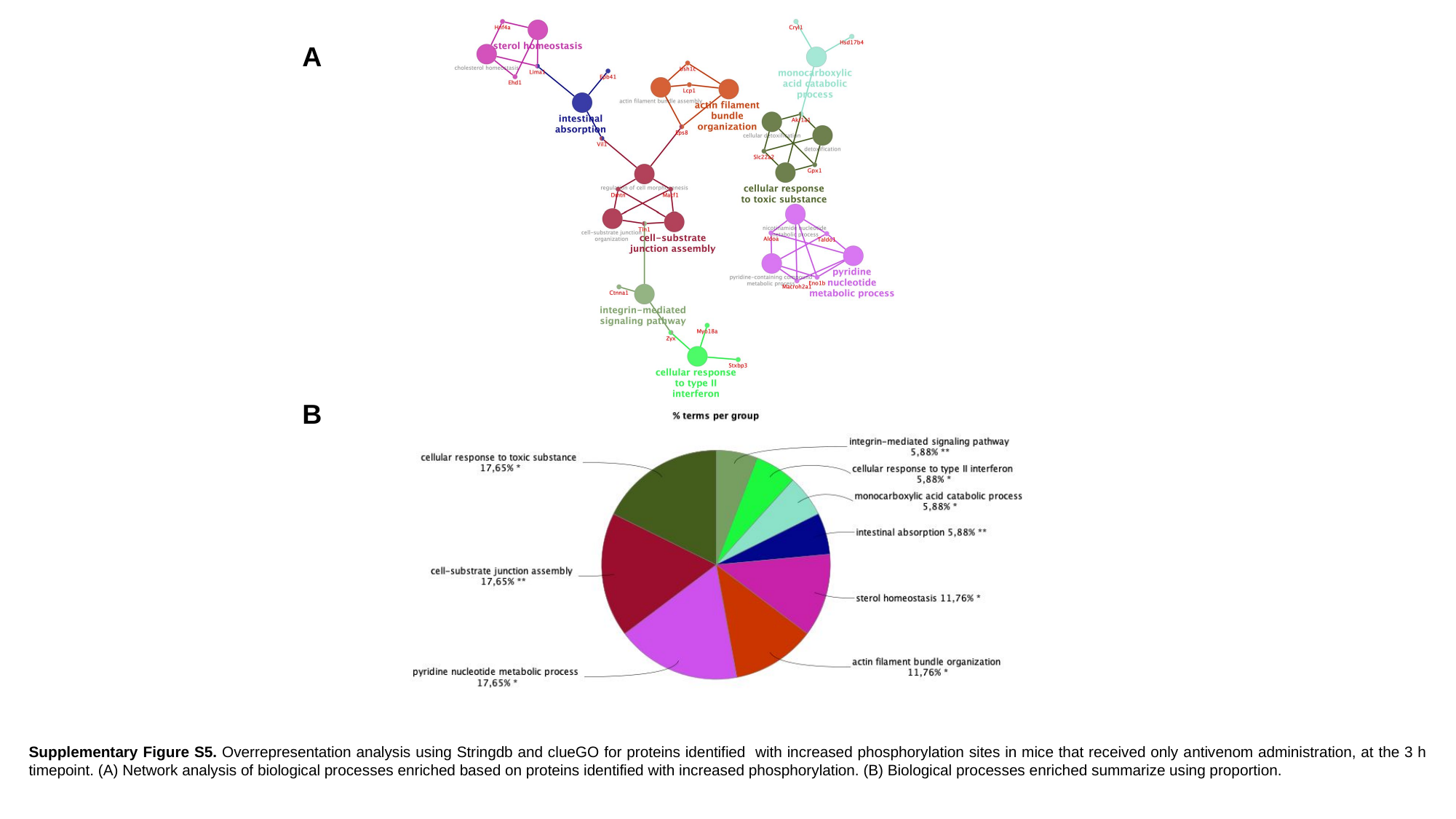

A
B
Supplementary Figure S5. Overrepresentation analysis using Stringdb and clueGO for proteins identified with increased phosphorylation sites in mice that received only antivenom administration, at the 3 h timepoint. (A) Network analysis of biological processes enriched based on proteins identified with increased phosphorylation. (B) Biological processes enriched summarize using proportion.

## Slide 6
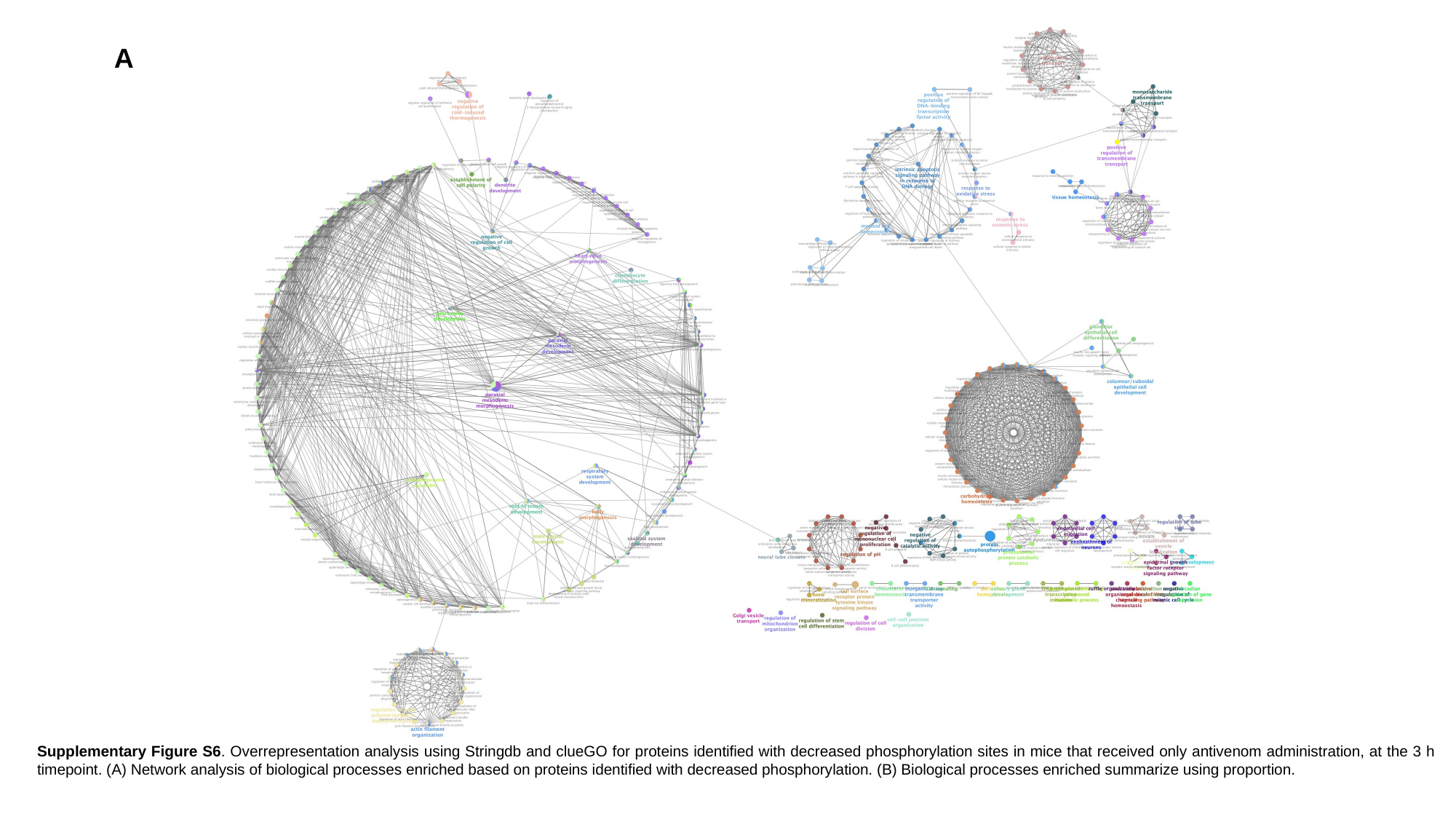

A
Supplementary Figure S6. Overrepresentation analysis using Stringdb and clueGO for proteins identified with decreased phosphorylation sites in mice that received only antivenom administration, at the 3 h timepoint. (A) Network analysis of biological processes enriched based on proteins identified with decreased phosphorylation. (B) Biological processes enriched summarize using proportion.

## Slide 7
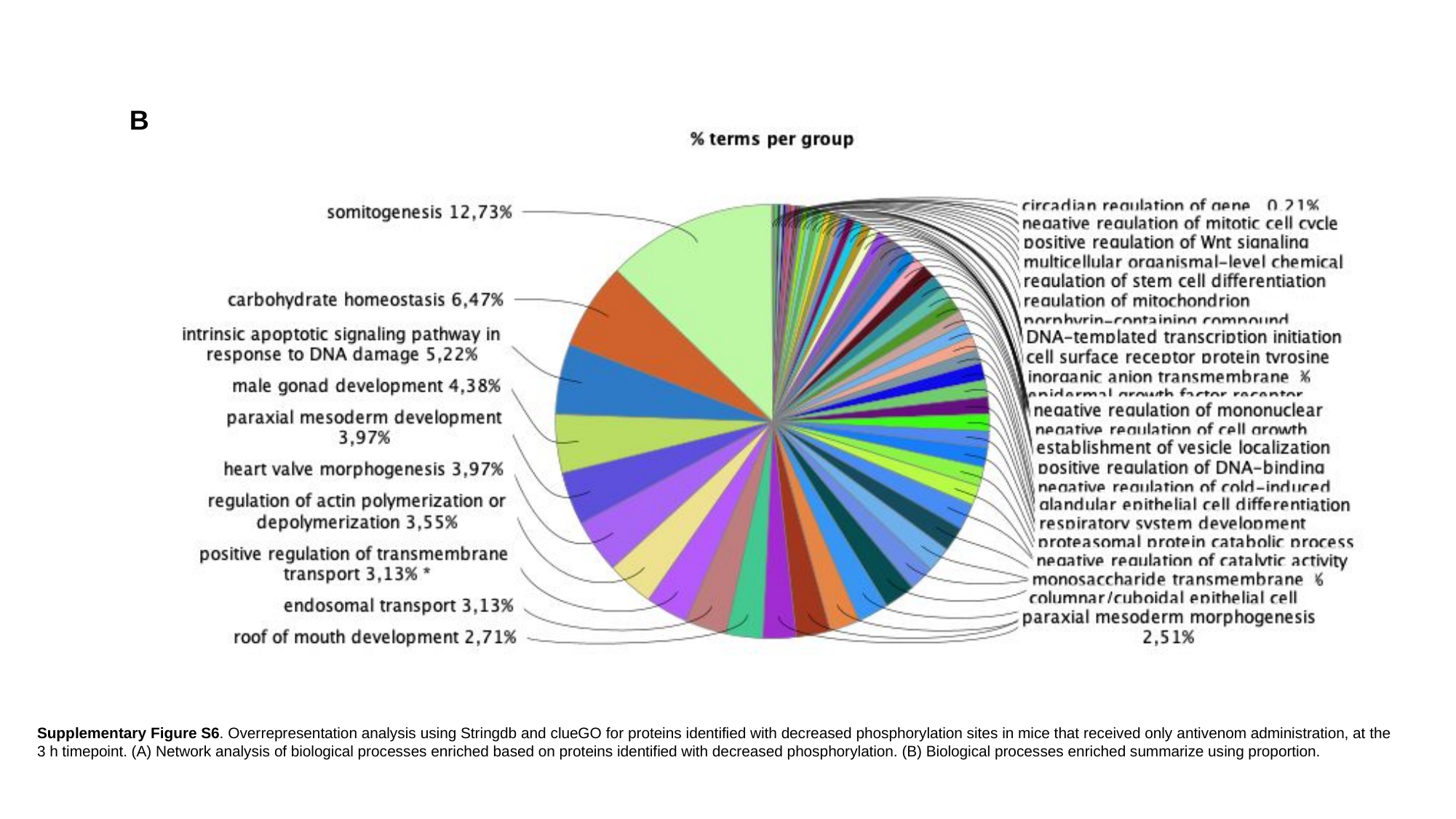

B
Supplementary Figure S6. Overrepresentation analysis using Stringdb and clueGO for proteins identified with decreased phosphorylation sites in mice that received only antivenom administration, at the 3 h timepoint. (A) Network analysis of biological processes enriched based on proteins identified with decreased phosphorylation. (B) Biological processes enriched summarize using proportion.
